# Exploring the impact of GSTM1 as a novel molecular determinant of survival in head and neck cancer patients of African descent

**DOI:** 10.1186/s13046-024-03127-3

**Published:** 2024-07-23

**Authors:** Fan Yang, Fanghui Chen, Chloe Shay, Georgia Z. Chen, Nabil F. Saba, Yong Teng

**Affiliations:** 1 Department of Hematology and Medical Oncology, School of Medicine, Winship Cancer Institute, Emory University, Atlanta, GA 30322 USA; 2 Wallace H. Coulter Department of Biomedical Engineering, Georgia Institute of Technology & Emory University, Atlanta, GA 30322 USA

**Keywords:** GSTM1, Head and neck cancer, Genomic profiling, African descent, Racial disparities, Tumor tissue microarray

## Abstract

**Background:**

Blacks/African American (BAA) patients diagnosed with head and neck squamous cell carcinoma (HNSCC) have worse survival outcomes than White patients. However, the mechanisms underlying racial disparities in HNSCC have not been thoroughly characterized.

**Methods:**

Data on gene expression, copy number variants (CNVs), gene mutations, and methylation were obtained from 6 head and neck cancer datasets. Comparative bioinformatics analysis of the above genomic features was performed between BAAs and Whites. The expression pattern of GSTM1 was validated by immunohistochemistry using tumor tissue microarray (TMA). Effect of GSTM1 knockdown were assessed by cell proliferation, colony formation, and tumor development in an orthotopic mouse model. The changes in protein kinases were determined using the Proteome Profiler Human Phospho-Kinase Array Kit in HNSCC cells with or without GSTM1 knockdown.

**Results:**

We identified ancestry-related differential genomic profiles in HNSCC. Specifically, in BAA HNSCC, FAT1 mutations were associated with its gene expression, SALL3 gene expression correlated with its gene CNVs, and RTP4 gene expression showed an inverse correlation with its methylation. Notably, GSTM1 emerged as a prognostic risk factor for BAA HNSCC, with high gene CNVs and expression levels correlating with poor overall survival in BAA patients. Immunohistochemistry results from newly developed in-house TMA validated the expression pattern of GSTM1 between BAA HNSCC and White HNSCC. In an orthotopic mouse model, GSTM1 knockdown significantly inhibited malignant progression in tumors derived from BAAs. In contrast, loss of GSTM1 did not affect the development of HNSCC originating in Whites. Mechanistically, GSTM1 knockdown suppressed HSP27 phosphorylation and β-catenin in BAA HNSCC cells, but not in White HNSCC cells. This differential effect at least partially contributes to tumor development in BAA patients.

**Conclusion:**

This study identifies GSTM1 as a novel molecular determinant of survival in HNSCC patients of African descent. It also provides a molecular basis for future research focused on identifying molecular determinants and developing therapeutic interventions to improve outcomes for BAA patients with HNSCC.

**Supplementary Information:**

The online version contains supplementary material available at 10.1186/s13046-024-03127-3.

## Background

Head and neck squamous cell carcinoma (HNSCC) encompasses a group of cancers arising from the epithelia of the upper aerodigestive tract, accounting for devastating malignancies associated with severe morbidity, high mortality, and limited treatment options [1–4]. Racial survival disparities in HNSCC have long been recognized between White and Black/African American (BAA) patients [5–8]. For example, BAA patients with HNSCC have a disproportionally increased tumor burden and a lower 5-year survival (29.3-31.0%) than Whites (54.7-59.0%) [7, 10–14]. Compared with Whites, the survival disadvantage in BAA HNSCCC patients is most likely attributed to more advanced stage and higher rates of metastasis and treatment resistance [7–9]. A retrospective study also reported a lower survival rate of BAAs than Whites with localized HNSCC [15]. Racial disparities commonly result from a complex interplay of factors, including treatment inequalities, socioeconomic status, and environment [7–9]. In addition, genetic factors of HNSCC patients largely contribute to the survival disparities observed between Whites and BAAs [16]. Chaudhary *et al* performed analysis using the cancer genome atlas (TCGA) and cancer digital archive of HNSCC patients (1992-2013) and found BAA patients with HNSCC had a higher frequency of mutations compared to Whites, particularly in the key driver genes: *P53*, *FAT1*, *CASP8* and *HRAS* [10]*.*

HNSCC in BAAs also exhibited lower intratumoral infiltration of effector immune cells (including CD8^+^, resting memory CD4^+^ and activated memory CD4^+^ T cells) with shorter survival than in Whites [14], suggesting specific systemic therapeutic candidates for the treatment of BAA patients. It is worth noting that our national policies are aimed to reduce racial disparities in cancer screening, diagnosis, treatment, and mortality (Pub Law) [17]. However, a complete understanding of how genetic aberrations drive differential tumor phenotypes and treatment responses in White and BAA patients with HNSCC remains largely unknown. As such, translation of these genomic, transcriptomic, and proteomic findings into targeted and individualized therapeutic strategies for treating HNSCC patients has been limited.

By integrating bioinformatics, genomics, transcriptomics, and proteomics data, we performed a comprehensive analysis of distinct molecular profiles between Whites and BAAs with HNSCC. Our study provides a molecular basis for racial disparities in HNSCC, which will inspire the development of more effective targeted therapies capable of prolonging the life of BAA patients with HNSCC to reduce the disparities in treatment outcomes.

## Materials and methods

### BAA and White HNSCC patient cohort with gene expression

Genomic (RNA-seq: transcriptome profiling and gene expression quantification) and clinical data from TCGA HNSCC cohort (*n* = 523) were collected (https://portal.gdc.cancer.gov/repository). HNSCC cases were sub-grouped into BAAs (*n*=47), Whites (n = 448), Asians (*n* = 11), American Indians or Alaska Natives (*n* = 2) and others (*n* = 15). The HNSCC subtype, clinical course, gene mutations, overall survival (OS), race and tumor mutational burden (TMB) annotation were obtained from cBioPortal datasets (https://www.cbioportal.org/datasets) for HNSCC (TCGA, PanCancer Atlas). The gene expression matrix was constructed via Perl software with the gene symbol and the gene raw counts of the expression matrix were normalized *via* log2 (counts+1). Gene high and low expression were distinguished based on the Z-scores. The average (α) of the log_2_ (counts+1) and standard deviation (σ) values were counted for each gene of the samples. Z-scores value = (β-α)/σ, where β is the log_2_ (counts+1) of gene. Z-score > 2 indicates high gene expression, while z-score < -2 indicates low expression. Gene high and low expression were defined as 20% of samples with the highest gene expression and 20% of samples with the lowest gene expression, respectively. The prognosis of differential genes was calculated by R package and shown by Kaplan-Meier survival curves and overall P value.

### Mutation analysis for BAA and White HNSCC using TCGA and other datasets

SNVs data (data type: masked somatic mutation) and clinical data of a total of 554 White patients and 54 BAA patients were obtained from 6 head and neck cancer datasets, including TCGA dataset (523 cases); Broad Institute of MIT and Harvard dataset (74 cases); Johns Hopkins University dataset (32 cases); University of Texas MD Anderson Cancer Center dataset (40 cases); Memorial Sloan Kettering Cancer Center (MSK) dataset (151 cases); and National University of Singapore dataset (56 cases). We calculated the top 50 most frequently mutated genes for BAA HNSCC and White HNSCC, respectively. By comparing the mutation frequencies, we focused on the top 20 gene mutations (e.g., *TP53, CSMD3, MUC16, LRP1B, KMT2D, FAT1* and *PIK3CA*) and their correlations between gene expression and survival *via* cBioPortal datasets. However, only TCGA dataset includes gene expression data. To investigate the correlation between gene mutations and gene expression, the total SNVs matrix and gene expression matrix (normalized gene counts, log_2_ (counts+1)) of HNSCC from TCGA were merged by R software with the same samples and gene symbols. The correlation of TMB score with gene expression was evaluated *via* Spearman’s rank correlation.

### Gene CNVs analysis for BAA and White HNSCC in TCGA and MSK datasets

Gene CNVs data were obtained from the HNSCC TCGA dataset and MSK dataset with ‘Masked copy number segment’ which includes 552 White cases and 54 BAA cases. The CNVs matrix was integrated *via* Perl software with gene symbol and copy number (-2: decreased two or more copies; -1: decreased one copy; 0: normal copy number; 1: increased one copy; 2: increased two or more copies). We counted the CNV frequencies for each gene (e.g., *AKR1C1, AKR1C2, AKR1C3, CAMK1D, IL17A, IL17F* and *SALL3*) and their differences were compared via Chi-square test (*P* < 0.05) in BAAs vs. Whites, and chromosomal mapping of differential genes was also performed. To explore the correlation between gene CNVs and gene expression level, the gene expression data was only available from the TCGA dataset; we integrated the CNVs matrix and gene expression matrix from TCGA *via* R software with same samples and gene symbol. The correlations were assessed by Spearman’s rank correlation (*P* < 0.05).

### Methylation analysis for BAA and White HNSCC in TCGA and MSK datasets

Gene methylation data were downloaded from TCGA and MSK HNSCC dataset with ‘Methylation Beta Value’ and ‘Illumina Human Methylation 450k BeadChip platform’, including 448 White cases and 47 BAA cases. The gene methylation matrix was integrated *via* Perl software with gene symbol and methylation beta value. Differences in gene methylation between BAA HNSCC and White HNSCC were calculated by Wilcox_test [*P* < 0.05, logFC= log_2_ (gene means of BAAs) - log_2_ (gene means of Whites)] in R software. BAA gene means were calculated as (*i*_1_+*i*_2_+…+*i*_48_)/BAA number (47) and White gene means as (*i*_1_+*i*_2_+…+*i*_452_)/White number (448); *i*_n_ is the methylation beta value of the nth sample. To investigate the correlation between gene methylation level and gene expression, the total gene methylation matrix (methylation beta value) and gene expression matrix of HNSCC were merged by R software with the same samples and gene symbols. The ‘limma’ and ‘MethylMix’ packages were performed to integrate standardized methylation matrix and gene expression matrix and construct mixed models, and calculate the differential methylation between BAAs and Whites via Wilcoxon rank test, and correct the *P*-values. The correlations between gene methylation site and gene expression were also counted. Correlations were assessed by Spearman’s rank correlation (*P* < 0.05).

### Pathway analysis

HNSCC-related genes and their functional enrichment and pathways were analyzed via GO annotation and KEGG dataset. *P* values were calculated using 20,603 protein-coding genes as reference sets with Fisher Exact test, and the false discovery rate (FDR) adjusted q-values were counted with the Benjamini-Hochberg procedure. The normalized gene expression matrix containing all samples from BAA and White patients was processed to obtain the gene expression ‘.gct’ file and sample ‘.cls’ file for GSEA. All samples were divided into BAA and White patient groups. The gene sets database was selected with ‘c2.cp.kegg.v2022.1.Hs.symbols.gmt’, the phenotype labels were selected ‘BAAs vs. Whites’, and the chip platform was selected ‘Human gene symbol with remapping MSigDB.v2022.1.Hs.Chip’. After successfully running the analysis, we obtained a rank gene list, positive and negative snapshot of enrichment results, and GSEA reports for BAAs and Whites.

### Patient survival analysis

Gene expression data from a total of 606 HNSCC patients were obtained using integrated 6 head and neck cancer datasets, of which 54 BAAs and 552 Whites had OS data, while 47 BAAs and 448 Whites had disease-specific survival (DSS) and progression-free survival (PFS) data. The prognosis of BAAs and Whites was evaluated *via* the ‘survival’ and ‘survminer’ package in R, respectively. The OS of 489 HNSCC patients by age and HPV (+/-) was calculated to evaluate the correlation between HNSCC_HPV- and HNSCC_HPV+ in TCGA dataset. All patients were divided into two subgroups, HNSCC_HPV+ (*n* = 73) and HNSCC_HPV- (*n* = 416), and the OS of BAA and White patients in TCGA HNSCC_HPV- or HNSCC_HPV+ was analyzed to evaluate the difference.

### Tumor tissue microarray (TMA) construction and immunohistochemistry (IHC)

All clinical specimens were obtained with written informed consent from the patients and collected under an Emory IRB-approved protocol (IRB00003208). Cases of interest were selected from the clinical archive of Emory University Department of Pathology from years 2015 to 2022. These tumor tissues were collected from biopsies or surgical specimens prior to any treatment with informed consent and reviewed by at least two pathologists at Emory to reconfirm the diagnosis. Tumor-related patient information including age, gender, TNM stage, pathological stage, lymph node metastasis, smoking history, HPV status, treatment(s) and survival status has been entered Emory WinDATA under HIPAA regulations and was available for use. Diagnostic hematoxylin and eosin (H&E) slides from each case were reviewed by an experienced pathologist to identify areas of tumor and normal tissue in the donor blocks. Tissue cores (1.5mm) from the indicated areas of the donor blocks were transferred to a recipient TMA block using a Pathology Devices TMArrayer semi-automated tissue microarrayer. Three cores from each selected tumor were placed randomly within a single TMA along with cores from representative normal tissue. A map was generated linking each core to its donor block and associated clinical information. Sections from the TMA blocks were cut at 5 μm for downstream analysis. IHC of TMA was performed as previously described [18, 19]. In brief, IHC was performed by incubating the sections with the primary antibody against GSTM1 (1:800, Novusbio, 1H4F2). Immunoreactivity was visualized using the DAB Detection kit (Vector laboratories, Burlingame, CA) and counterstained with hematoxylin. Slides were dehydrated, mounted, and scanned using the Olympus Nanozoomer whole slide scanner (Olympus, Center Valley, PA). Negative controls included non-specific polyclonal rabbit antibody at 2 μg/ml (Abcam, Cambridge, MA). The final immunoreactivity score was examined by an experienced pathologist and two investigators who were blind to pathological information by using the German semi-quantitative scoring method as we previously described [20, 21]. Each specimen was scored for intensity (no staining = 0; weak staining = 1; moderate staining = 2; strong staining = 3) and for extent of stained cells (0% = 0; 1-24% = 1; 25-49% = 2; 50-74% = 3; 75-100% = 4). Consecutive sections were stained with hematoxylin and eosin (H&E) to help localize cancer tissues and adjacent normal epithelium. The signal index (SI) of each tissue was calculated as the product of the intensity score multiplied by the extent score. The SI was then correlated with the corresponding patient survival data, stratified into BAA and White patients.

### Cell lines

Human HN12 cells were a kind gift from Dr. Andrew Yeudall in 2016 and maintained in our lab [22]. SCC9 cells were purchased the American Type Culture Collection (ATCC, Manassas, VA). JHU029 cells were obtained from Dr. David Sidranski at Johns Hopkins University (Baltimore, MA). All cell lines were used for experiments before passage 10 and cultured in complete DMEM medium (Thermo Scientific, Waltham, MA) containing 10% FCS (Biological Industries), 2 mM L-glutamine (Biological Industries) and 1% pen-strep (Gibco, USA) at 37°C in a humidified incubator supplied with 5% CO_2_. All cell lines were routinely screened for mycoplasma contamination by MycoAlert Mycoplasma Detection Kit (Lonza).

### Western blot

Whole-cell lysates were solubilized in cell lysis buffer (Cell Signaling Technology; Cat# 9803) with protease inhibitors (Sigma-Aldrich). Protein concentrations were assayed using BCA Protein Assay (Thermo Fisher; Cat# 23225) and 30 mg total protein was loaded per lane onto 8% SDS-PAGE (70 min, 120 V). Gels were transferred to Immobilon PVDF membranes (Millipore). Membranes were blocked in TBST containing 5% Bovine Serum Albumin (BSA) prior to incubation with appropriate primary and secondary antibodies. Blots were incubated in Immobilon Western HRP substrate (Millipore). Chemiluminescence was captured using Amersham Imager 600 (GE). Antibodies specific for the following proteins were used for western blot: GSTM1 (Novus biologicals; Cat# NBP2-22186), β-Catenin (Cell Signaling Technology; Cat#37447), HSP27 (Cell Signaling Technology; Cat# 2402), p-HSP27 (Ser82) (Cell Signaling Technology; Cat# 2401), and β-actin (Sigma Aldrich; Cat# A5316).

### Gene modifications

The pLKO.1-puro shRNA against GFP (shGFP) and shRNAs targeting the GSTM1 gene (shGSTM1-1, shGSTM1-2 and shGSTM1-3) were purchased from Horizon Discovery (Waterbeach, UK). ViraPower Lentiviral Packaging Mix contains an optimized mixture of the three packaging plasmids (pLP1, pLP2, and pLP/VSVG) and was obtained from Invitrogen (Carlsbad, CA). Lentiviral shRNA plasmids, together with packaging plasmids were co-transfected into Lenti-Pac 293TA cells (GeneCopoeia, Rockville, MD) using Lipofectamine 3000 (Invitrogen, Carlsbad, CA) according to the manufacturer’s instructions. Two days after transfection, viral particles were harvested and transfected into HNSCC cells to generate stable knockdown cell lines. The efficacy of knockdown was evaluated by Western blot. In this study, if there is no specific mention, shGSTM1 refers to shGSTM1-1.

### Cell proliferation and clonogenic assays

Cell proliferation was determined by MTS Assay Kit (Abcam, Cambridge, UK). For clonogenic assays, GSTM1 knockdown or control HNSCC cells were seeded in six-well plates at a cell concentration of 1 × 10^3^ cells/well, and the plates were incubated at 37°C with 5% CO_2_. After incubation for 10 days, cells were fixed using 10% formaldehyde and stained using 0.4% crystal violet, and the number of colonies (>50 cells/colony) was counted.

### Phospho-kinase profiling

The Proteome Profiler Human Phospho-Kinase Array Kit (R&D Systems, Minneapolis, MN) was used to assess the changes in the relative levels of phosphorylation at 37 kinase phosphorylation sites and 2 related total proteins in HNSCC cells with or without GSTM1 knockdown. Briefly, 500 μg of fresh protein was diluted and incubated overnight with nitrocellulose membranes blotted with double spots for the indicated antibodies. Bound phospho-kinases were detected using a pan-phosphotyrosine antibody conjugated to horseradish peroxidase. Data were digitized and analyzed using ImageJ Fiji (version 1.2). Relative protein or phosphorylation levels of the indicated protein kinases were obtained by subtraction of background staining and normalization to positive controls on the same membrane.

### Animal studies

Six-week-old male NOD.Cg-*Prkdcscid Il2rgtm1Wjl/SzJ* (NSG) mice were purchased from Jackson Laboratory (Bar Harbor, ME). All animal experiments were approved by the Institutional Animal Care and Use Committee (IACUC) of Emory University. To generate an orthotopic tumor model to evaluate the role of GSTM1 in head and neck tumor development, 1×10^6^ GSTM1 knockdown and control HNSCC cells were suspended in 100 μl of PBS/Matrigel (3:1) and injected into buccal mucosa of NSG mice as we previously described [22]. Tumor dimensions were serially measured with electronic calipers, and tumor volume was calculated by the formula of Volume = length × width^2^ × 1/2.

### Statistical analysis

Statistical software GraphPad Prism 9 (San Diego, CA) was used for all statistical analyses. Experimental values are expressed as mean ± standard error of the mean (SEM). For comparison between two groups, statistical analysis was performed using unpaired Student’s *t*-test. Differences were considered statistically significant when *P* < 0.05.

## Results

### Overall survival in BAA patients is significantly shorter than in White patients with HPV-unrelated HNSCC

**Fig. 1:**
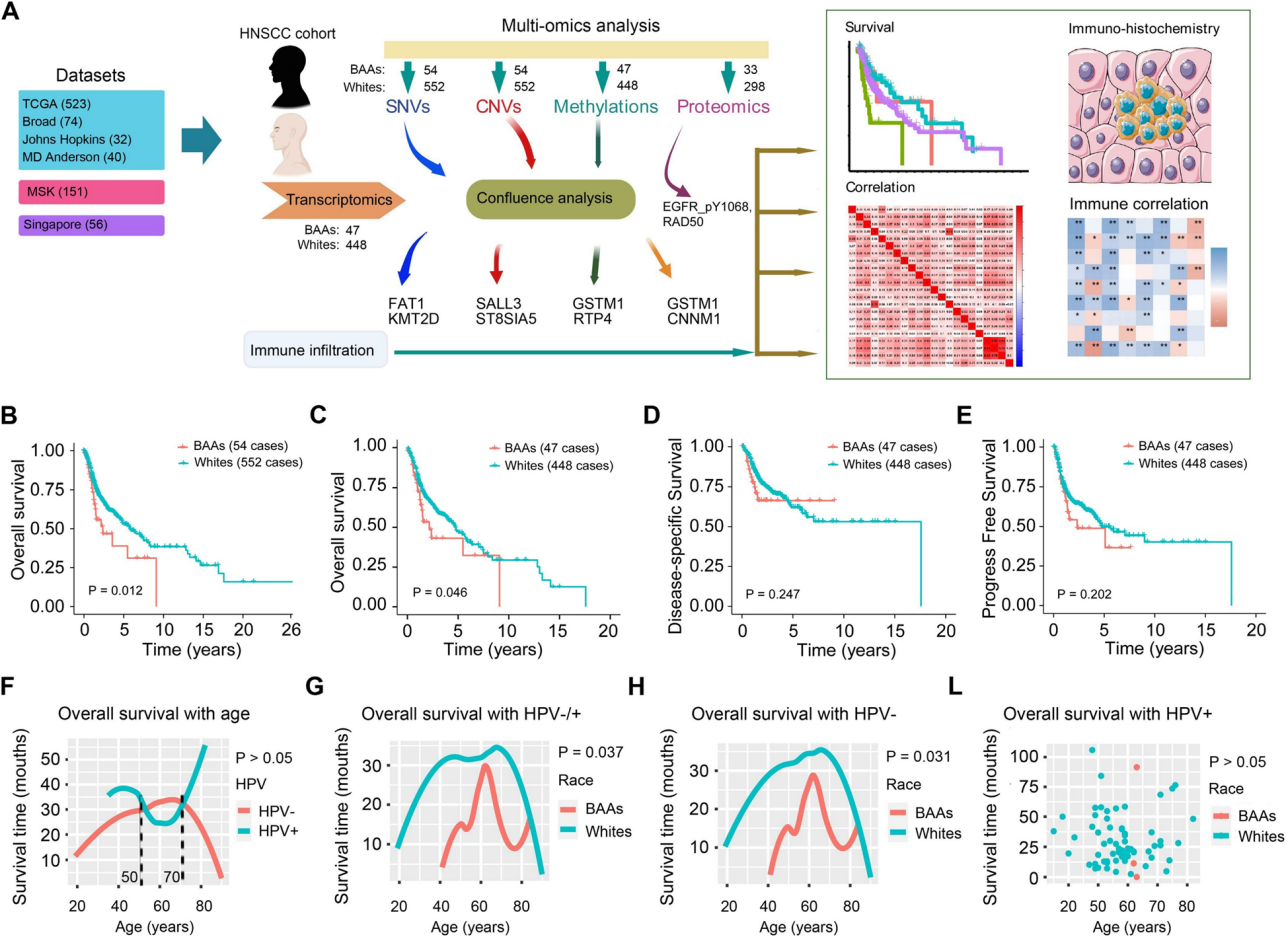
Survival analysis for BAA and White patients with HNSCC. **A** Flowchart of multi-omics analysis for BAA HNSCC and White HNSCC in TCGA HNSCC cohort. **B** OS analysis for BAA HNSCC vs. White HNSCC in 606 patients (*P* < 0.05). **C** OS analysis for BAA HNSCC vs. White HNSCC in 495 patients (*P* < 0.05)). **D** DSS analysis for BAA HNSCC vs. White HNSCC in 495 patients (*P* > 0.05). **E** PFS analysis for BAA HNSCC vs. White HNSCC in 495 patients (*P* > 0.05). **F** OS analysis for total HNSCC patients based on patient age and HPV infection status in 495 patients. **G** OS analysis for BAA HNSCC and White HNSCC with patients’ age and HPV-/+ status in 495 patients (*P* < 0.05). **H** OS analysis for BAA HNSCC vs. White HNSCC based on patient age and HPV- status in 495 patients (*P* < 0.05). **L** OS analysis for BAA HNSCC vs. White HNSCC based on patient age and HPV status in 495 patients (*P* > 0.05)

In this study, we focused on multi-omics analysis by integrating the data of single nucleotide variants (SNVs), copy number variants (CNVs), gene methylations, transcriptomics, and proteomics for HNSCC in BAAs and Whites (Fig. 1A). From 6 head and neck cancer datasets, we obtained data from 54 BAA HNSCC patients and 552 White HNSCC patients. We first integrated the clinical data from these patients, including overall survival (OS), progression-free survival (PFS) and disease-specific survival (DSS), to evaluate the prognostic difference between BAAs and Whites with HNSCC. This analysis showed that OS in BAAs with HNSCC was significantly shorter than in Whites with HNSCC (*P* < 0.05, Fig. 1B). Analysis of the patients’ prognosis from TCGA showed that OS of BAA patients was significantly shorter than in Whites (*P* < 0.05), with no difference in PFS and DSS between BAAs and Whites (Fig. 1C-E). We also analyzed the association of OS with other major clinical characteristics, including HPV infection status, sex and tumor stage, using the datasets from TCGA HNSCC cohort. Although OS of HNSCC patients is also significantly associated these clinical characteristics (Supplementary Fig. S1), we focused more in the present study on distinct genomic profiles that contribute to racial disparities.

We next analyzed OS of HNSCC patients by age and HPV infection status using fit analysis to better understand the genetic difference between BAA and White HNSCC from TCGA. Interestingly, HPV infection had an age effect on OS in HNSCC patients, which was accentuated at ages 50-70 (Fig. 1F). In addition, OS in BAAs with HNSCC was significantly shorter than in White patients (*P* = 0.037) (Fig. 1G). More particularly, poorer OS was found in BAAs with HPV-unrelated (-) HNSCC relative to Whites with HPV(-) HNSCC (median of 20.56 months for BAAs and 31.17 months for White patients, *P* = 0.031). Lower OS was also observed in BAA patients compared with the same age White patients (Fig. 1H). However, only a small fraction of BAAs had HPV-related (+) HNSCC, and there was no noticeable difference in OS between BAAs and Whites with this subtype of tumors (Fig. 1L). These observations indicate that BAAs with HNSCC have a worse prognosis than Whites, especially for HPV(-) tumors.

### Differential genetic mutation spectrum in BAA and White HNSCC

**Fig. 2:**
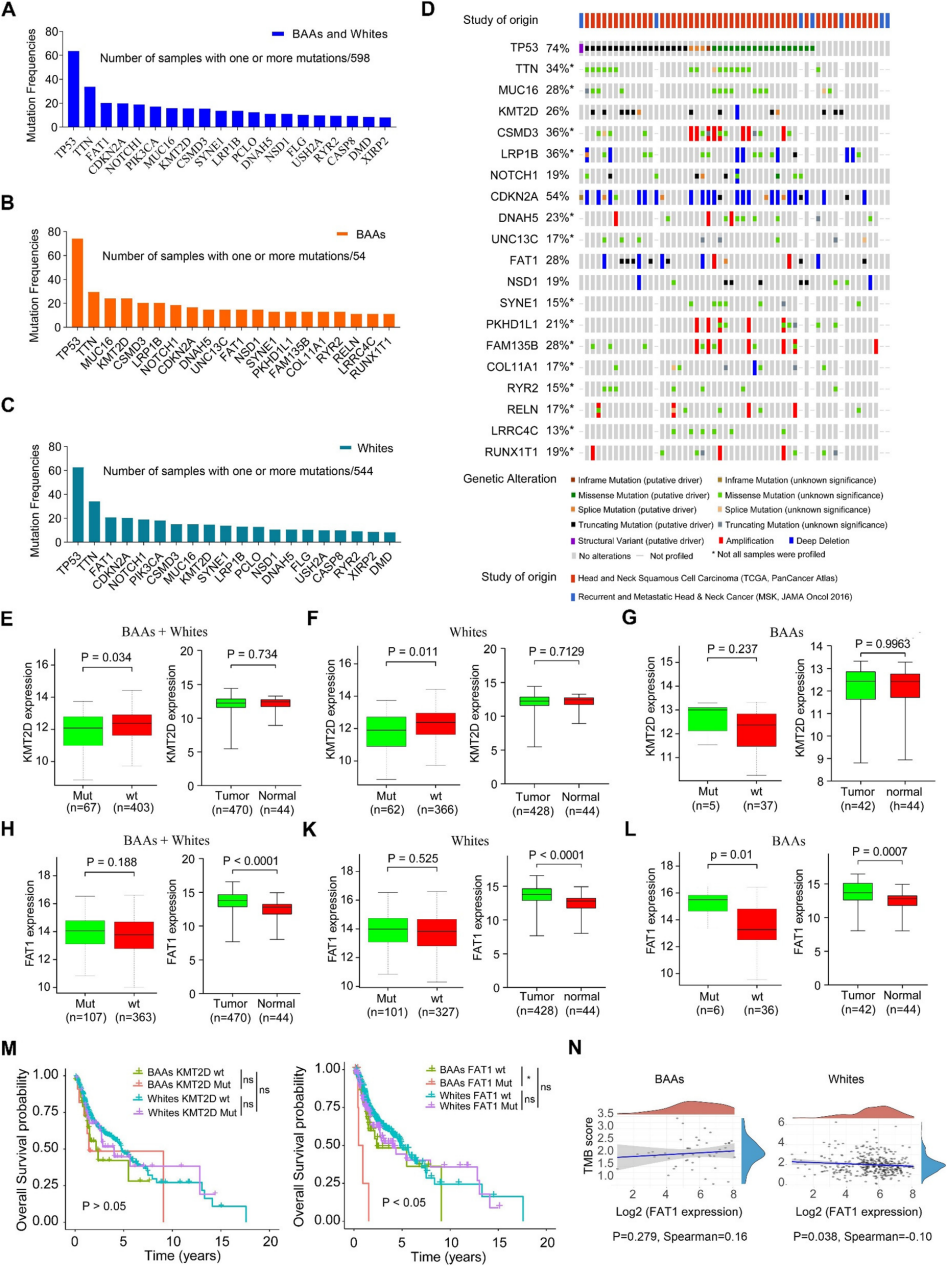
Somatic mutation analysis for BAA and White HNSCC in TCGA and other datasets. **A** Top 20 most frequently mutated genes among BAA and White HNSCC cases. **B** Top 20 most frequently mutated genes in BAA HNSCC. **C** Top 20 most frequently mutated genes in White HNSCC. **D** Correlation analysis of gene mutations, genetic alterations (amplification and deletion) and mRNA expression for the top 20 mutated genes in all 54 BAA HNSCC. **E**-**L** Correlation analysis between gene mutations and expression for KMT2D and FAT1 genes. **M** Overall survival analysis of KMT2D and FAT1 gene mutations in BAAs and Whites with HNSCC. **N** Correlation analysis between gene expression and TMB score for the FAT1 gene in BAA and White HNSCC. ns, not significant, *P<0.05 and **P<0.01

To identify the representative number of genetic mutations of HNSCC patients, we compared SNVs between BAA and White HNSCC using SNV data in TCGA and MSK cohorts. Total mutation frequencies (Fig. 2A-C) and genetic alterations, such as missense mutation, amplification, splice mutation, and deep deletion for each gene among BAAs (Fig. 2D) and Whites with HNSCC were calculated. Here we focused on the top 20 mutated genes in BAA HNSCC vs. White HNSCC (Supplementary Table S1). In BAA HNSCC, the 5 most commonly mutated genes were TP53, TTN, MUC16, KMT2D, and CSMD3 (Fig. 2B and D), while in White HNSCC, TP53, TTN, FAT1, CDKN2A, and NOTCH1 were the 5 most commonly mutated genes (Fig. 2C and supplementary Fig. S2). Strikingly, the frequencies of gene mutations did not correlate with their corresponding gene SNVs in both BAA and White HNSCC (Fig. 2A-C). The top 20 most mutated genes with the highest SNV frequencies in BAA HNSCC were CDKN2A (37%), CSMD3 (17%), FAM135B (14.9%), LRP1B (14.9%), FAT1 (10.6%), PKHD1L1 (10.6%), and the frequencies of 14 other genes were less than 10.0%. In White HNSCC, the top 20 genes with the highest SNV frequencies were CDKN2A (25.5%), PIK3CA (13.6%), LRP1B (12.4%), FAT1 (5.3%), and the frequencies of 16 other genes were less than 5.0%. Correlation analysis revealed that KMT2D mutation was significantly associated with KMT2D expression in HNSCC regardless of race (Fig. 2E-G). In contrast, FAT1 mutation was significantly associated with its gene expression in BAA HNSCC (Fig. 2H-L). In addition, FAT1 mutation is a risk factor for BAAs with HNSCC as FAT1 mutation was positively associated with poor OS in BAAs (Fig. 2M). There was no correlation between mutant FAT1 expression and TMB in BAA HNSCC. A weak negative correlation was found between mutant FAT1 expression and TMB in White HNSCC (Fig. 2N).

### SALL3 gene expression is associated with its gene CNVs in BAA HNSCC

**Fig. 3:**
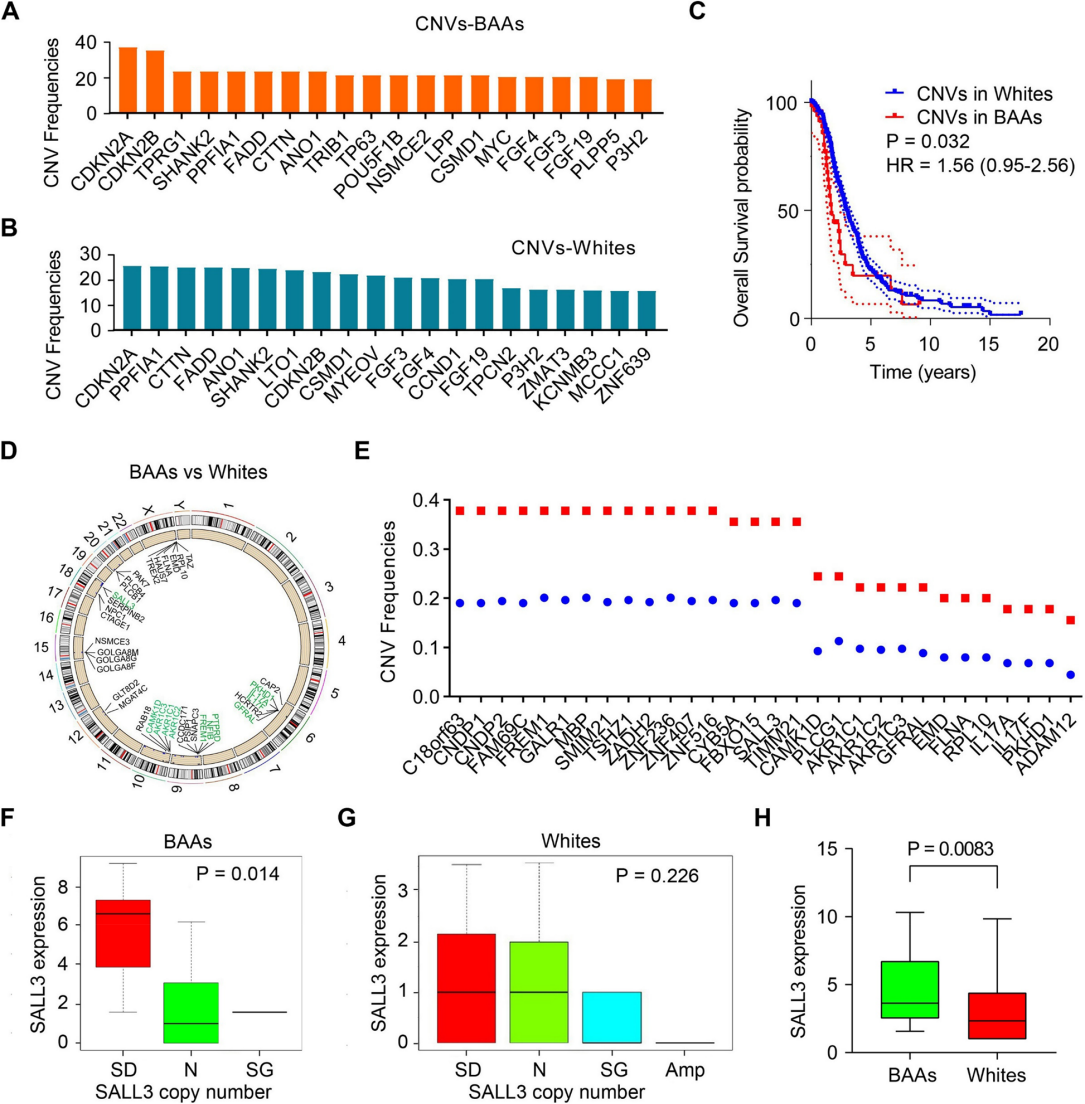
Gene CNV analysis for BAA and White HNSCC in TCGA HNSCC cohort. **A** Top 20 genes with highest CNV frequency among BAA HNSCC. **B** Top 20 genes with highest CNV frequency among White HNSCC. **C** Overall survival analysis of gene CNVs in BAAs and Whites with HNSCC. **D** Localization of the genes in chromosome (Red, SALL3 and ST8SIA5 CNVs are associated with gene expression; green: belong to top 30) and **E** Top 30 genes with highest CNV frequency among BAA vs White HNSCC. **F**-**G** Correlation of SALL3 gene CNVs with its expression in (**F**) BAA HNSCC and (**G**) White HNSCC. (H) Expression of SALL3 in BAA HNSCC vs. White HNSCC. SD: single deletion; N: normal; SG: single gain; Amp: amplification

The 20 genes with highest CNV frequencies were much different between BAA HNSCC and White HNSCC (Fig. 3A and B). In BAA HNSCC, CDKN2A, CDKN2B, TPRG1, SHANK2, and FADD were the 5 genes with highest CNV frequency (Fig. 3A). In White HNSCC, CDKN2A, PPFIA1, CTTN, FADD, and ANO1 were the 5 genes with highest CNV frequency (Fig. 3B). Prognostic analysis of TCGA HNSCC cohort revealed that alterations in CNVs affect OS in BAAs with HNSCC more significantly than in White patients (*P* = 0.032) (Fig. 3C). Hazard ratio (HR) analysis suggests that CNVs could increase risk in BAA HNSCC compared with White HNSCC (HR = 1.558) (Fig. 3C). Using Chi-square test, we identified 119 genes (*P* < 0.05) with variable CNVs in BAA HNSCC compared with White HNSCC (Fig. 3D), and these genes include AKR1C1, AKR1C2, AKR1C3, IL17A, IL17F and SALL3 (Fig. 3D-E). Correlation analysis showed that the copy number of the SALL3 gene was strongly associated with its gene expression in BAA HNSCC, but not in White HNSCC (Fig. 3F-H). Moreover, cluster analysis of gene expression in BAA HNSCC revealed that SALL3, C6orf15, NELL1 and GSTM1 are in the same cluster and there was a strong co-expression between SALL3 and GSTM1 genes (Spearman = 0.66, *P* = 0.00011) (Supplementary Fig. S3). Pathways enrichment analysis indicated that SALL3 is enriched in ‘Cell adhesion molecules’, ‘Neutrophil extracellular trap formation’ and ‘Salivary secretion’ signaling pathways in BAA HNSCC (Supplementary Fig. S4).

### Ancestry-related differences in DNA methylation

**Fig. 4:**
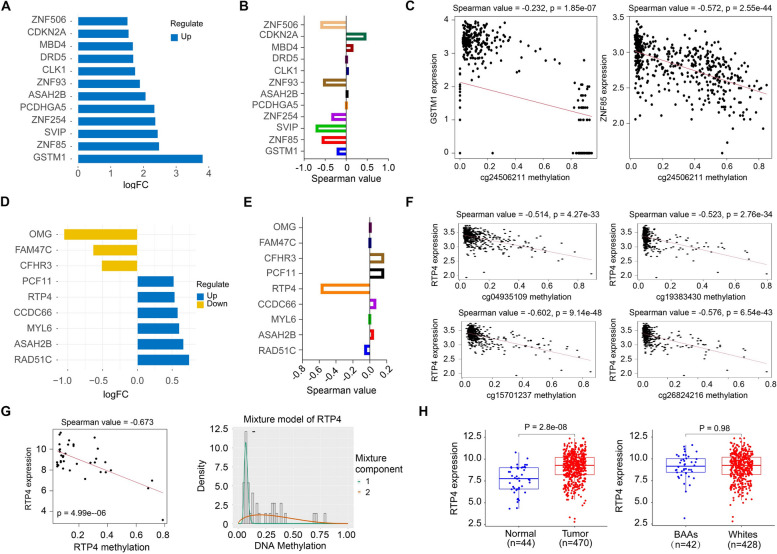
Gene methylation analysis for BAA and White HNSCC in TCGA HNSCC cohort. **A** Genes with the highest methylation level in BAA HNSCC vs. normal. **B** Correlation between methylation of those genes listed in (**A**) and their expression in BAA HNSCC. **C** Correlation between methylation at the methylation sites of GSTM1 (cg24506221) and ZNF85 (cg11416076) genes and their gene expression in BAA HNSCC. **D** Genes with the greatest differential methylation level in BAA HNSCC vs. White HNSCC. **E** Correlation between methylation of those genes listed in (**D**) and their gene expression in BAA HNSCC. **F** Correlation between RTP4 gene methylation at four methylation sites (cg04935109, cg19383430, cg15701237 and cg26824216) and its gene expression in BAA HNSCC. **G** Correlation between methylation of RTP4 gene and its gene expression in BAA HNSCC (left), and methylation level analysis of RTP4 gene in BAA (green line) and White (orange line) HNSCC (right). (H) RTP4 gene expression in BAA and White HNSCC

DNA methylation is commonly regarded as a silencing mechanism. To understand the impact of gene methylation on racial disparities in HNSCC, we first evaluated the gene methylation alterations between HNSCC and normal controls. This analysis identified 97 genes with significant methylation alterations in HNSCC compared with normal controls. GSTM1, ZNF85, SVIP, ZNF254 and PCDHGA5 were the top 5 genes with aberrant DNA methylation in HNSCC (Fig. 4A). Correlation between the methylation of these genes and their gene expression was calculated, which showed Spearman_GSTM1_ = -0.226, Spearman_ZNF85_ = -0.582, Spearman_SVIP_ = -0.727, Spearman_ZNF254_ = 0.354, and Spearman_PCDHGA5_ = -0.028 (Fig. 4B). Further correlation analysis for gene methylation sites and expression were also conducted. Higher methylation levels of nine methylation sites at the SVIP gene correspond to lower gene expression (Supplementary Fig. S5). Intriguingly, only one methylation site was significantly associated with gene expression in gene GSTM1 (cg24506221) and ZNF85 (cg11416076) in HNSCC (Fig. 4C). Next, we evaluated the gene methylation alterations in BAA HNSCC compared with White HNSCC. This analysis identified 23 significant genes carrying significant methylation alterations in BAA HNSCC. These genes include RAD51, ASAII2B, MYL6, CCDC66 and RTP4 (Fig. 4D), of which only RTP4 methylation was moderately correlated with its gene expression (Spearman value = -0.584, *P* =2.679e-44) (Fig. 4E). There are four methylation sites (cg049350109, cg15701237, cg19383430 and cg26824216) in the RTP4 gene strongly associated with its gene expression (Fig. 4F). Moreover, 5 of 33 methylation driven genes, including RTP4, IER5, POU3F1, SIT1 and TYMP, were identified whose methylation levels were inversely correlated with their gene expression in BAA HNSCC (Supplementary Fig. S6). A strong inverse correlation (Spearman = -0.673, *P* = 4.991e-06) was observed between RTP4 methylation and its expression in BAA HNSCC (Fig. 4G). In addition, RTP4 methylation level was significantly lower in BAA HNSCC than in White HNSCC (Fig. 4G). Although RTP4 expression is significantly upregulated in HNSCC, no significant difference was found between BAA and White HNSCC (Fig. 4H).

### GSTM1 is a potential prognostic risk factor for BAAs with HNSCC

**Fig. 5:**
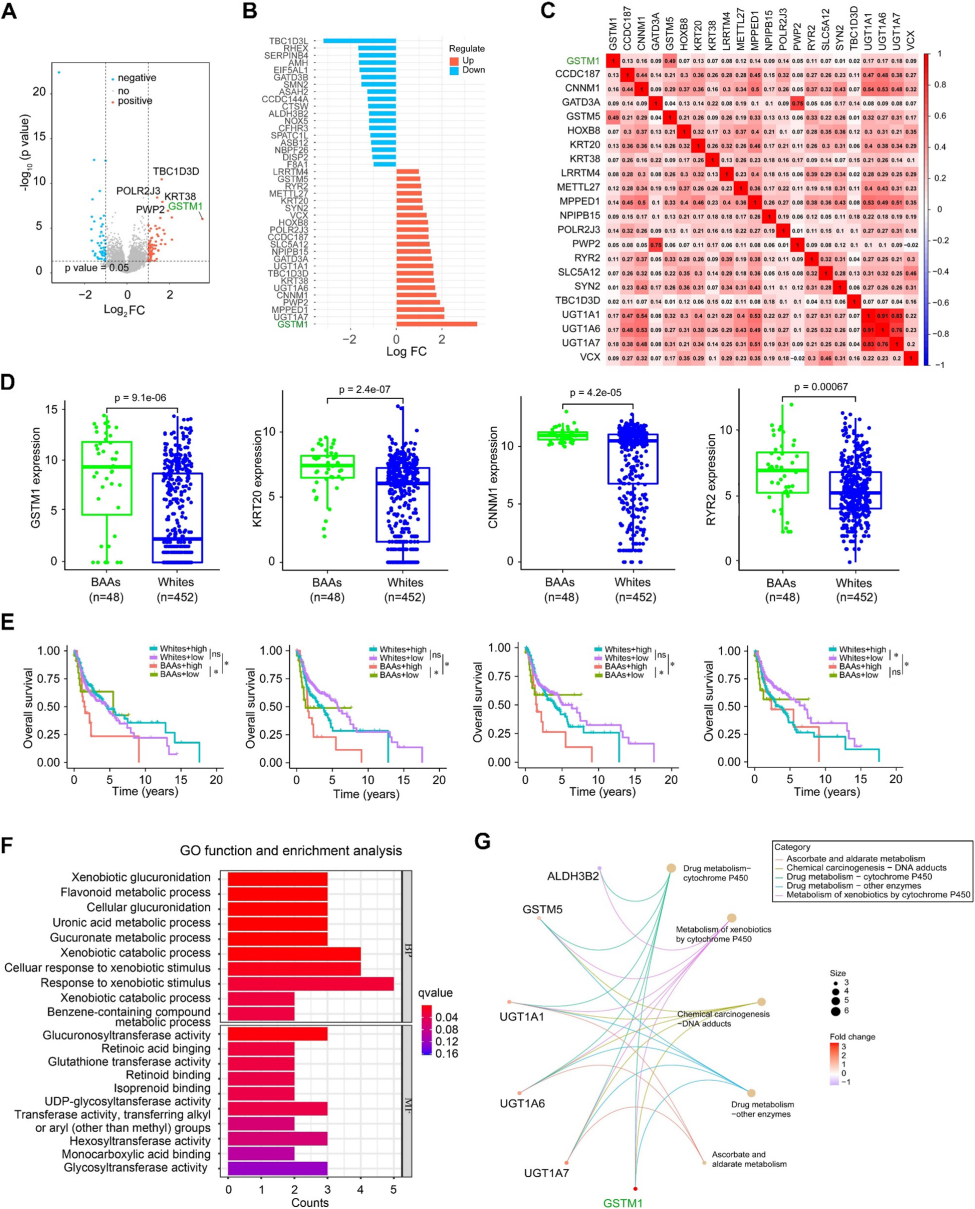
Transcriptomics analysis for BAA and White HNSCC in TCGA HNSCC cohort. **A** Volcano plots of differentially expressed genes in BAA HNSCC vs. White HNSCC. **B** Heatmap showing differentially expressed genes in BAA HNSCC vs. White HNSCC. **C** Correlation between expression of GSTM1 and 21 other genes that are mostly upregulated in BAA HNSCC vs. White HNSCC. **D** Gene expression of GSTM1, KRT20, CNNM1 and RYR2 between BAA HNSCC and White HNSCC. **E** Overall survival analysis of GSTM1, KRT20, CNNM1 and RYR2 gene expression in BAA HNSCC vs. White HNSCC. **F** GO function and enrichment analysis of GSMT1 gene in BAA HNSCC. **G** Pathways enrichment analysis of GSTM1 gene in BAA HNSCC. ns, not significant, *P<0.05 and **P<0.01

To identify differentially expressed genes (DEGs) in BAA HNSCC vs. White HNSCC, the TCGA HNSCC dataset was divided into BAA and White groups and gene expression analysis was performed. This analysis identified 160 DEGs (Fig. 5A), of which 22 genes, including GSTM1, PWP2 and MPPED1, were significantly upregulated in BAA HNSCC compared with White HNSCC (*P* < 0.05, logFC >1) (Fig. 5B). From this list, GSTM1 was the most upregulated gene in BAA HNSCC, and its expression level was moderately associated with GSTM5 among the top 22 most upregulated genes (Fig. 5C). We next analyzed the association of DEGs with the survival rate in BAAs and Whites with HNSCC. Among the top 20 upregulated (Supplementary Fig. S7) and downregulated genes (Supplementary Fig. S8), the expression levels of four genes (GSTM1, KRT20, CNNM1, and RYR2) upregulated in BAA HNSCC were positively associated with OS in these patients, but not in Whites (Fig. 5D-E). These data suggest that these four genes could be developed into risk factors for predicting prognosis of BAAs with HNSCC.

Strikingly, simultaneous evaluation of the association between GSTM1 expression and the five major clinical characteristics (race, sex, HPV infection status, age and tumor stage) in the TCGA HNSCC cohort. This analysis revealed that GSTM1 expression is only significantly correlated with race (Supplementary Fig. S9). Moreover, GO and KEGG pathway analysis showed that GSTM1 is significantly enriched in the ‘xenobiotic metabolic process’ and ‘xenobiotic glucuronidation’ terms of biological processes (BP) (Fig. 5F) and ‘metabolism of xenobiotics by cytochrome p450’and ‘drug metabolism-cytochrome p450’ pathways (Fig. 5F-G).

**Fig. 6:**
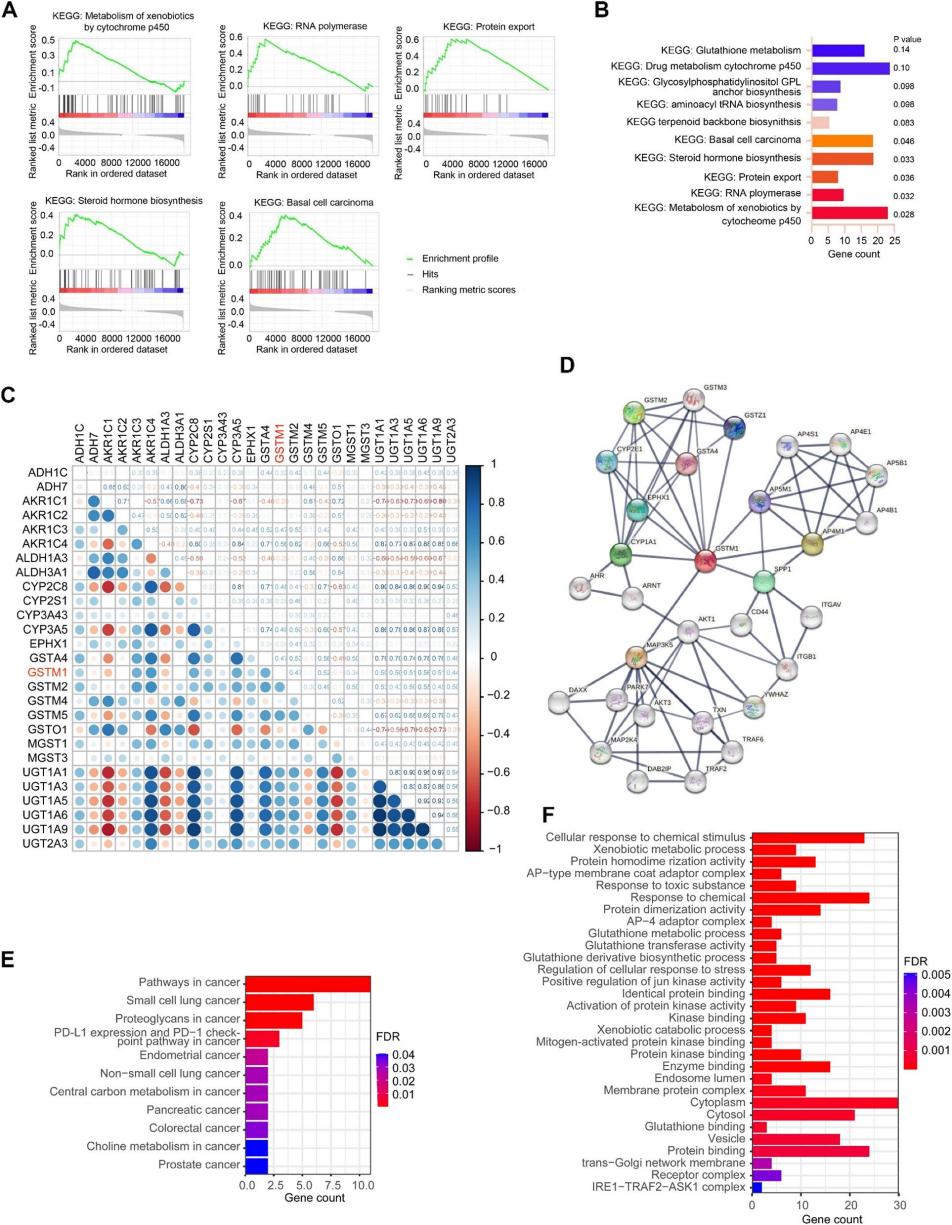
Pathway enrichment and protein-protein interaction (PPI) analysis for differentially expressed genes in BAA HNSCC in TCGA HNSCC cohort. **A**, **B** GSEA and KEGG pathway enrichment analysis of the top dysregulated pathways in BAA HNSCC vs. White HNSCC. **C** Correlation expression among genes in the ‘Metabolism of xenobiotics by cytochrome p450’ pathway in BAA HNSCC. **D** Functional protein association networks of the GSTM1 pathway analyzed by STRING10.5 (local clustering coefficient=0.717, PPI enrichment p value=7.46e-10). **E** Enrichment analysis of GSTM1 in human cancers based on the results from STRING10.5. **F** Pathways enrichment analysis of GSTM1 in human based on the results from STRING10.5

To better understand the signaling alterations between BAA and White HNSCC, we performed GSEA and pathway enrichment analysis based on gene expression in BAA and White HNSCC. This analysis revealed GSTM1 as one of the top 50 most upregulated genes in BAA HNSCC vs. White HNSCC (Supplementary Fig. S10), and indicated that five pathways, including ‘metabolism of xenobiotics by cytochrome p450’ pathway, were the most upregulated pathways in BAA HNSCC compared with White HNSCC (Fig. 6A-B). Interestingly, GSTM1 was enriched in the ‘metabolism of xenobiotics by cytochrome p450’ pathway (Fig. 5F), suggesting that GSTM1 may be the molecular determinant to activate this pathway in BAA HNSCC. Furthermore, the correlation between GSTM1 and genes involved in ‘metabolism of xenobiotics by cytochrome p450’ was analyzed, showing that GSTM1 is highly correlated with CYP3A5, CYP2C8 and AKR1C4 (Spearman value ≥ 0.6) and moderately associated with ADH1C, AKR1C3, GSTM2, GSTM5, UGT1A1, UGT1A3, UGT1A5, UGT1A6, UGT1A9 and UGT2A3 in BAA HNSCC (0.4 < Spearman value < 0.6) (Fig. 6C). The String10.5 analysis was performed to determine the inner linkage among GSTM1, and its interacting partners based upon enrichment pathway analysis. As shown in Fig. 6D, MAP3K5, CYP1A1, AP5M1, APRM1, EPHX1 and SPP1 are major effectors of GSTM1 signaling, in which other GST family members GSTA4 and GSTZ1 participate, with high confidence (interaction score = 0.7). The network protein functional enrichment was also calculated and significantly enriched in ‘pathways in cancer’, ‘small cell lung cancer’, ‘proteoglycans in cancer’ and ‘PD-L1 expression and PD-1 checkpoint pathway in cancer’ terms (Fig. 6E) and ‘xenobiotic metabolic process’ (Fig. 6F).

**Fig. 7:**
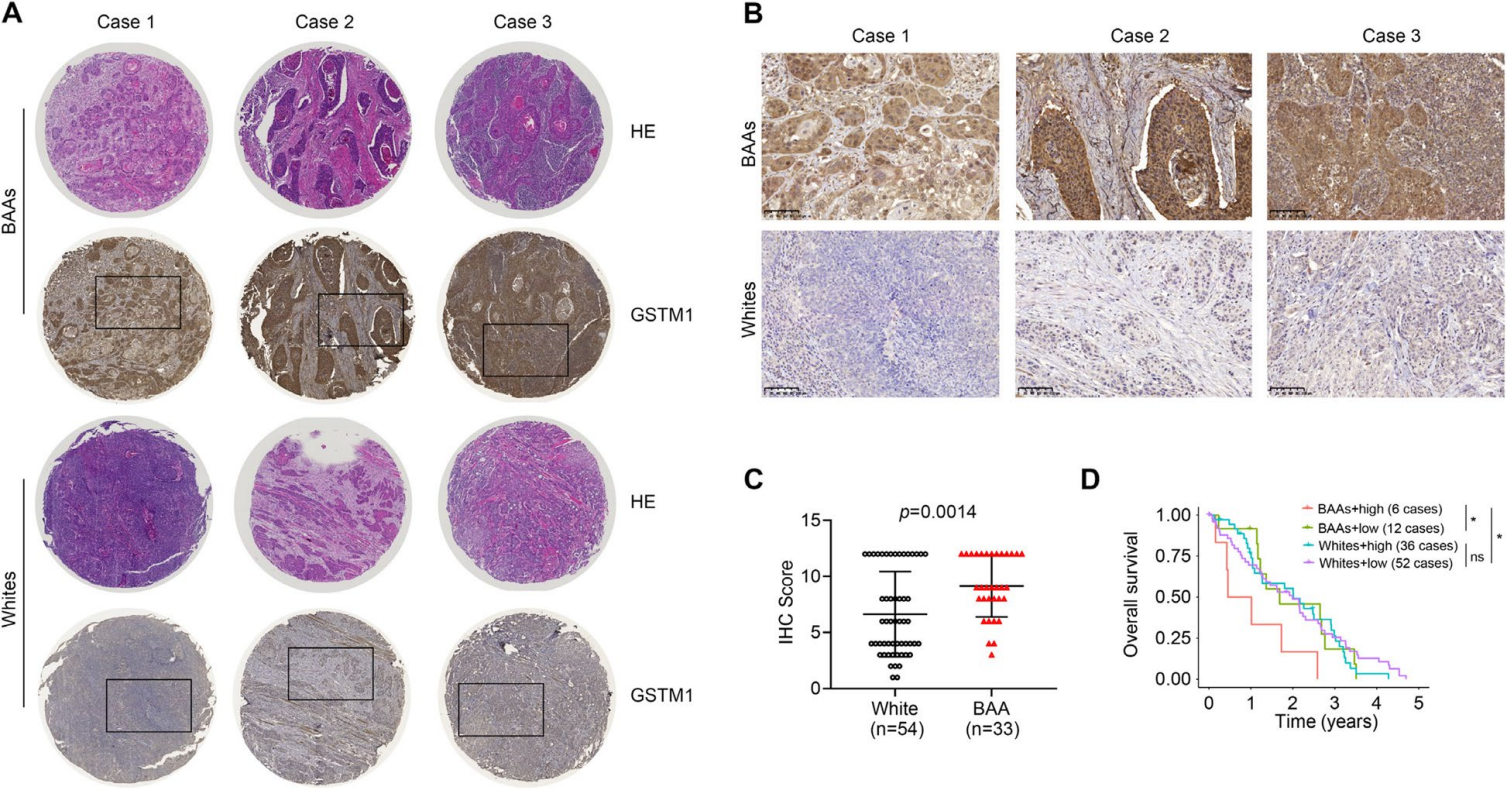
Immunohistochemistry analysis of GSTM1 protein levels in BAA and White HNSCC using in-house-made TMA. **A**, **B** Representative GSTM1 immunostaining in BAA and White HNSCC. Images with low and high magnification are shown in A and B, respectively. **C** Quantitative IHC score of GSTM1 in BAA (*n*=18) and White (*n*=88) HNSCC. **D** Overall survival analysis based on HNSCC patients with low (below median) vs. high (at or above median) GSTM1 staining. Survival data are stratified into BAAs and Whites

To validate the expression pattern of GSTM1 at protein levels, in-house-made TMA containing 18 BAA HNSCC cases and 88 White HNSCC cases was used for IHC. This analysis showed remarkably increased levels of GSTM1 in primary BAA HNSCC tissues compared with White HNSCC tissues (Fig. 7A-C). Notably, higher levels of GSTM1 were significantly associated with lower OS in BAAs, but not in Whites (Fig. 7D), suggesting GSTM1 may confer the development and progression of BAA HNSCC.

### Loss of GSTM1 in BAA-derived HNSCC cells suppresses tumor growth

**Fig. 8:**
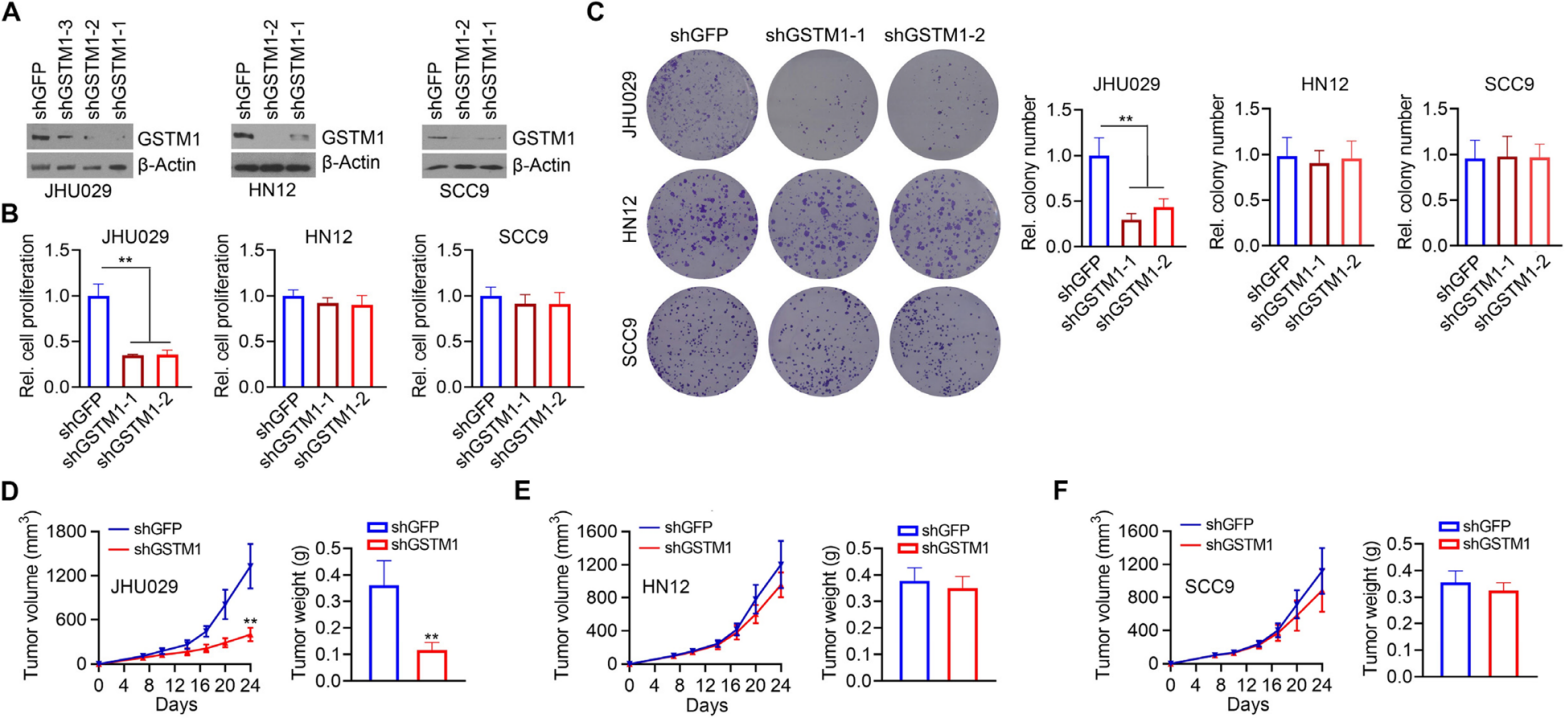
Knockdown of GSTM1 in HNSCC cells derived from BAA ancestry leads to tumor regression. **A** The knockdown efficiency of different GSTM1-targeting shRNAs (shGSTM1-1, shGSTM1-2, and shGSTM1-3) in three HNSCC cell lines determined by Western blot. **B** Effect of GSTM1 knockdown on HNSCC cell proliferation determined by MTS assay after 72 hours of cell culture. **C** Effect of GSTM1 knockdown on HNSCC cell colony formation determined by clonogenic assays after 10 days of cell culture. Representative images and quantitative data are shown in the left and right panels, respectively. **D**-**F** Effect of GSTM1 knockdown on tumor growth in an orthotopic tumor mouse model. Growth curve and weight of JHU029 tumors, HN12 tumors and SCC9 tumors are shown in (**D**), (**E**) and (**F**), respectively. *P<0.05, **P<0.01

The strong clinical significance of GSTM1 found in BAA HNSCC patients led us to investigate the specific functions of GSTM1 in HNSCC cells originating from BAAs. JHU029 cells were obtained from a BAA HNSCC patient, while HN12 and SCC9 were derived from individuals of White ethnicity [23]. We then depleted GSTM1 in JHU029, HN12 and SCC9 cells using lentiviral shRNAs. Two GSTM1-targeting shRNAs (shGSTM1-1 and shGSTM1-2) exhibited similar gene knockdown efficiency in these three cell lines (Fig. 8A). Interestingly, GSTM1 knockdown in JHU029 cells significantly reduced the potential in cell proliferation and colony formation compared with the knockdown control cells (Fig. 8B and C). In contrast, there was no noticeable difference in cell proliferation and colony formation in both HN12 and SCC9 cells with or without GSTM1 knockdown (Fig. 8B and C). In an orthotopic mouse model of HNSCC, mice implanted with GSTM1 knockdown JHU029 cells exhibited a substantial reduction in tumor volume and weight compared to the knockdown control group (Fig. 8D). This observation confirms the crucial role of GSTM1 in driving the growth of BAA head and neck tumors. Consistent with the in vitro data, no significant changes were noted in GSTM1 knockdown HN12 tumors when compared to the knockdown control tumors (Fig. 8E). A similar trend was observed in GSTM1 knockdown SCC9 tumors compared to the knockdown control tumors (Fig. 8F).

### GSTM1 plays a distinct role in BAA HNSCC cells by driving a different protein kinase regulatory network compared to White HNSCC cells.

**Fig. 9:**
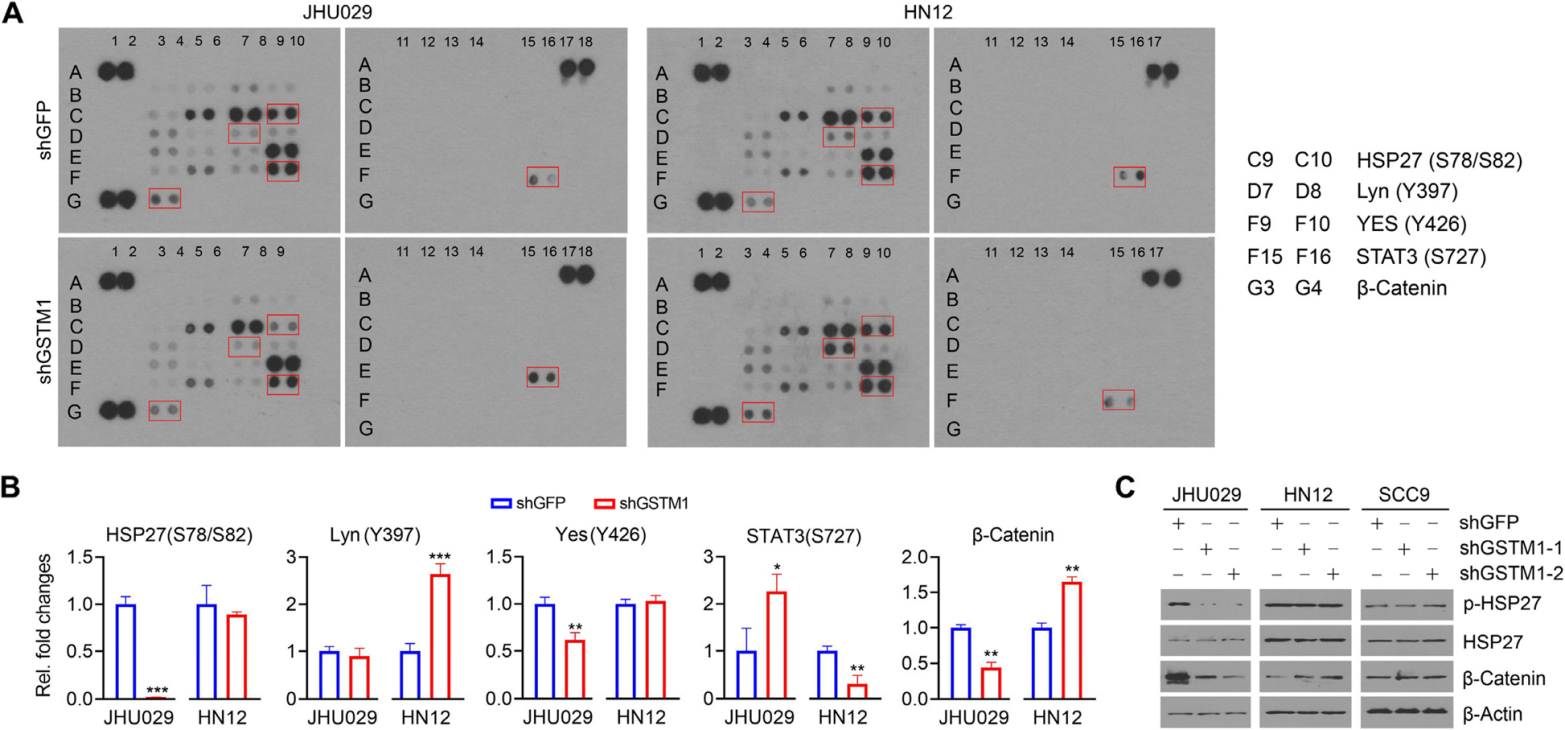
Knockdown of GSTM1 affects a different protein kinase regulatory network in BAA HNSCC cells compared to White HNSCC cells. **A**, **B** Effect of GSTM1 knockdown on the activation of protein kinases in JHU029 and HN12 cells determined using the Proteome Profiler Human Phospho-Kinase Array Kit. The protein kinases exhibiting different changes after GSTM1 knockdown in JHU029 and HN12 cells are framed in (**A**). The relative fold changes of these framed protein kinases upon GSTM1 knockdown are shown in (**B**). **C** Effect of GSTM1 knockdown on HSP27 phosphorylation levels and β-Catenin protein levels in three HNSCC cell lines determined by Western blot. *P<0.05, **P<0.01, ***P<0.001

Next, we investigated the molecular mechanisms behind the distinct role of GSTM1 between Black and White HNSCC cells. Since GSTM1 was not a transcription factor and likely functions through signaling modifications, we used the Proteome Profiler Human Phospho-Kinase Array Kit to analyze changes in protein kinases in HNSCC cells with and without GSTM1 knockdown. This analysis revealed distinct phospho-kinase profiles in JHU029 cells (derived from a BAA patient) and HN12 cells (derived from a White patient) upon GSTM1 knockdown (Fig. 9A and B). Specifically, when GSTM1 was depleted, JHU029 cells showed significantly downregulated levels of HSP27 and Yes phosphorylation and β-catenin protein, which were not observed in HN12 cells (Fig. 9A and B). In contrast, GSTM1 knockdown increased STAT3 phosphorylation levels in JHU029 cells, an effect opposite to that observed in HN12 cells when GSTM1 was knocked down (Fig. 9A and B). Decreased levels of HSP27 phosphorylation and β-catenin protein in GSTM1 knockdown JHU029 cells (vs. knockdown control cells) were confirmed by Western blot (Fig. 9C). Supporting the data from the Phospho-Kinase Array, Western blot analysis showed no change in HSP27 phosphorylation levels and increased β-catenin protein levels in HN12 and SCC9 cells (both derived from White patients) upon GSTM1 knockdown (Fig. 9C). These findings indicate that GSTM1 drives a different protein kinase regulatory network in BAA HNSCC cells compared to White HNSCC cells.

## Discussion

Racial disparities in HNSCC outcome and prognosis remain a major healthcare challenge. Accumulating evidence has shown that disparities persist even after adjustment for non-biological factors. Molecular analysis to identify disease-causing pathways in BAAs with HNSCC is limited. Mezghani et al., and Chaudhary et al., have reported some molecular subtypes present in BAAs with HNSCC and mutations related to immune regulation in this population [14, 24, 25]. Our multi-omics analysis further demonstrates the genetic variability and complexity in BAA compared to White HNSCC patients. Alterations in the p53 tumor suppressor gene in various types of cancers, particularly HNSCC, have been associated with poor prognosis. Consistent with other reports [24, 25], we found that TP53 is the most commonly mutated gene in HNSCC regardless of race. However, compared to Whites (62.7%), a relatively high frequency of TP53 mutation was found in BAAs (74.1%), which could be reflective of the lower incidence of HPV-related disease in this group. We did not find a good correlation of TP53 mutation with its expression in HNSCC regardless of race.

FAT1 is among the group of genes that is most frequently mutated in many cancers [26–29]. The highest mutation rate of FAT1 was found in HNSCC, ranking as the second most mutated gene after TP53 in this disease, and suggesting its critical role in HNSCC development and progression [2, 30, 31]. Our bioinformatics data demonstrate that FAT1 mutation correlates with its expression in BAA HNSCC. Moreover, FAT1 mutation is positively associated with poor OS in BAAs, suggesting it could serve as a risk factor for BAAs with HNSCC. The clinical implication of FAT1 mutations in BAAs highlights the need to fully understand FAT1 mutation sites and their functional alterations and develop agents that can specifically target the mutated FAT1 gene to improve health outcomes for BAA patients.

GSTs are a family of phase II detoxification enzymes that function to protect cellular macromolecules from attack by reactive electrophiles [32, 33]. The GST enzymes are grouped into three different classes including membrane-bound microsomal, mitochondrial, and cytoplasmic. There are at least 7 classes of cytoplasmic isoenzymes: alpha (A), mu (M), omega (O), pi (P), sigma (S), theta (T), and zeta (Z) [32–34]. They primarily carry out the catalytic detoxification of exogenous compounds. GSTP1 is the most studied GST isoform in different types of cancer, which has the potential to regulate AMPK/mTOR and MAPK signaling and facilitate protein synthesis and cell proliferation, respectively [35]. Like GSTP1, GSTM1 belongs to cytosolic GSTs and regulates the AMPK signaling pathway [36]. It was reported that GSTM1 inhibited dexamethasone-induced apoptosis in a lymphoblastic leukemia cell line [37]. Previous studies showed significant associations (OR=9.0, 95%CI; 1.4-9.5) of GSTM1 null genotype with HNSCC [37, 38]. In contrast, our analysis of TCGA data found only 4 cases of GSTM1 deep deletion (0.76%), suggesting the GSTM1 null genotype is not common in HNSCC. We further revealed that only GSTM1 of the GST family has higher CNVs in BAA HNSCC compared with White HNSCC, which is associated with its lower methylation and higher gene expression level.

Most importantly, higher levels of GSTM1 strongly correlate with lower survival in BAAs with HNSCC. We confirmed higher GSTM1 expression in BAA HNSCC compared with White HNSCC using our in-house-made TMAs. GSTs are thought to function in xenobiotic metabolism and play a role in susceptibility to cancer [32–34]. Pathway enrichment analysis of DEGs in BAA HNSCC vs. White HNSCC identified the involvement of GSTM1 in the ‘xenobiotic metabolic process’, ‘metabolism of xenobiotics by cytochrome p450’ and ‘drug metabolism-cytochrome p450’ pathways, suggesting that GSTM1 may be important in the treatment response of BAAs with HNSCC. Still, little is known about the underlying mechanisms by which GSTM1 expression is elevated in BAA HNSCC compared with White HNSCC, and further study to explore whether GSTM1-mediated chemoresistance and metastasis contribute to the worse prognosis of BAA patients is warranted.

Unfortunately, the availability of immortalized HNSCC cell lines from BAA patients is severely limited, with only a few accessible cell lines derived from this population [39–41]. The paucity of preclinical HNSCC models from the BAA population represents a significant barrier to research focused on this demographic. In this study, we only identified JHU029 as a BAA-derived HNSCC cell line, but it is critical to recognize that the differential tumor inhibitory effect of GSTM1 knockdown between JHU029 and two HNSCC cell lines derived from white individuals (HN12 and SCC9), may depend on the cellular context. Notably, our study on protein kinase profiling showed that GSTM1 knockdown significantly suppressed HSP27 phosphorylation and β-catenin protein levels in JHU029 cells but not in HN12 and SCC9 cells, suggesting that GSTM1 maybe contribute to HNSCC development and progression in BAAs by driving different protein kinase-mediated signaling pathways compared to White HNSCC. However, these observations should also be validated in a broader range of HNSCC cell lines derived from BAA and White individuals. To ensure a comprehensive understanding of the distinct role of GSTM1 in the ancestral disparity observed in patients, it is imperative to develop more diverse and representative preclinical models. While immortalized cell lines offer experimental tractability, they are genetically unstable, poorly reflect HNSCC heterogeneity, and lack representation of the tumor microenvironment (TME). Recent advances in the conditional reprogramming (CR) method have established a robust panel of HNSCC tumor cultures using a Rho kinase inhibitor (Y-27632) and co-culture with irradiated fibroblast feeder cells (J2 strain) for indefinite tumor cell survival [39]. CR cultures can further establish 3D organoids and patient-derived xenograft (PDX) models, providing a translational research model that incorporates patient and tumor diversity. We are currently engaged in efforts to establish CR culture systems using surgical specimens obtained from both BAA and White patients with HNSCC. This initiative aims to further investigate the role of GSTM1. In addition, an in-depth dissection of the regulatory network of GSTM1 is needed to identify the specific mechanism responsible for the upregulation of GSTM1 in BAA HNSCC cells.

### Supplementary Information

Supplementary Material 1.

## Data Availability

The datasets used and/or analyzed during the current study are available from the corresponding author on reasonable request.

## Abbreviations

BAAs: Black/African Americans
CNVs: Copy number variations
DSS: Disease-specific survival
FAT1: FAT atypical cadherin 1
FDR: False discovery rate
GSTM1: Glutathione S-transferase mu 1
H&E: Hematoxylin and eosin
HNSCC: Head and neck squamous cell carcinoma
IHC: Immunohistochemistry
OS: Overall survival
PFS: Progress free survival
SALL3: Spalt like transcription factor 3
SNVs: Single nucleotide variants
TCGA: The cancer genome atlas
TMB: Tumor mutational burden
TMA: Tissue microarray
