# Supplementary material for: Exploring the impact of GSTM1 as a novel molecular determinant of survival in head and neck cancer patients of African descent
Source: J Exp Clin Cancer Res. 2024 Jul 23;43:203. doi: 10.1186/s13046-024-03127-3 (PMC11264416; doi:10.1186/s13046-024-03127-3)
Supplement: Supplementary file 1 — Supplementary Material 1. [file 13046_2024_3127_MOESM1_ESM.docx]

**Supplemental Information for**

**Exploring the impact of GSTM1 as a novel molecular determinant of survival in head and neck cancer patients of African descent**

Yang et al

**Correspondence:** Yong Teng, [yong.teng@emory.edu](mailto:yong.teng@emory.edu)

This PDF file includes:

Supplementary Figures (Figure S1-S10)

Supplementary Table S1

**Figure S1.** **Correlation analysis between the major clinical features and patient overall survival (OS) in TCGA HNSCC cohort.** (A) Correlation between HPV infection status and OS in patients with HNSCC. (B) Correlation between sex and OS in patients with HNSCC. (C) Correlation between tumor stage and OS in patients with HNSCC.


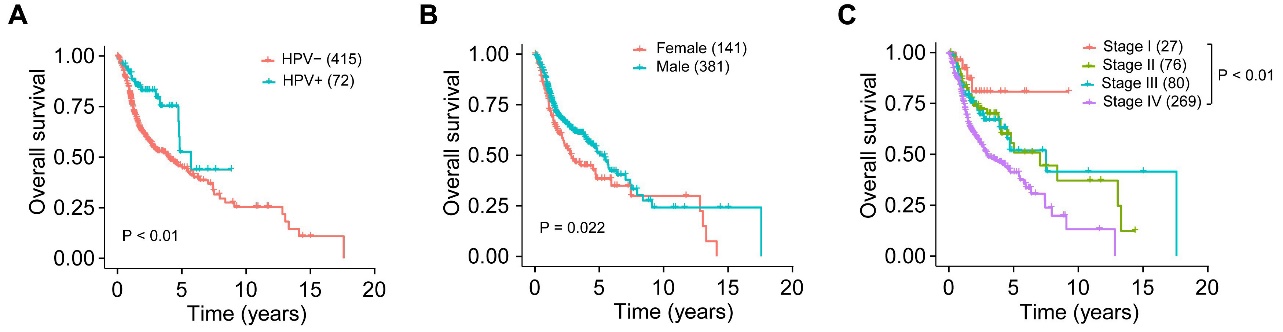


**Figure S2.** **Correlation analysis of gene mutations, CNVs, genetic alterations (amplification and deletion) and mRNA expression for top 20 mutated genes in White HNSCC based on TCGA HNSCC dataset.**


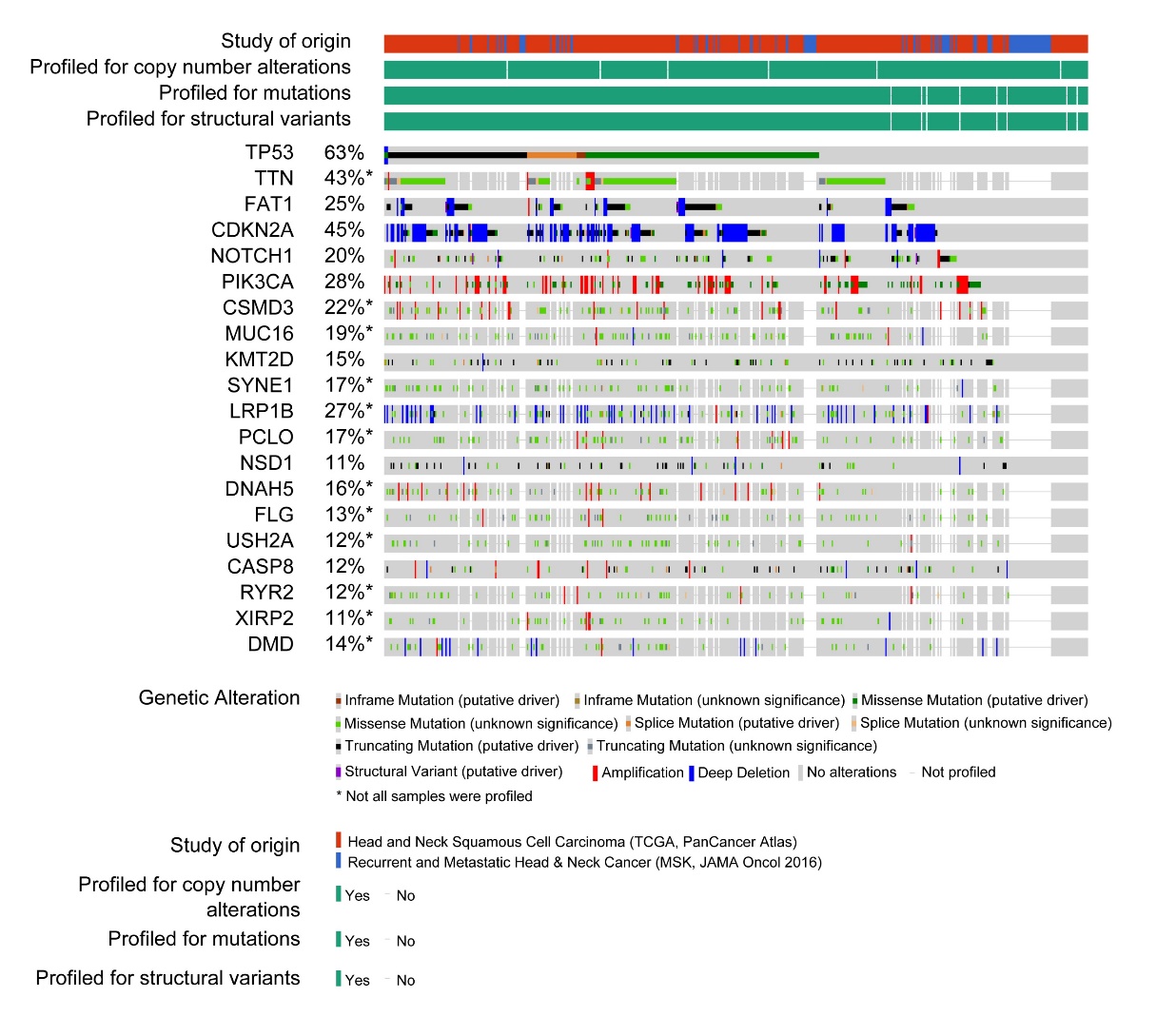


**Figure S3. A heatmap of the top 30 most variable genes with SALL3 high or low expression in BAA HNSCC.** GSTM1 expression correlates with SALL3 expression in BAA HNSCC.


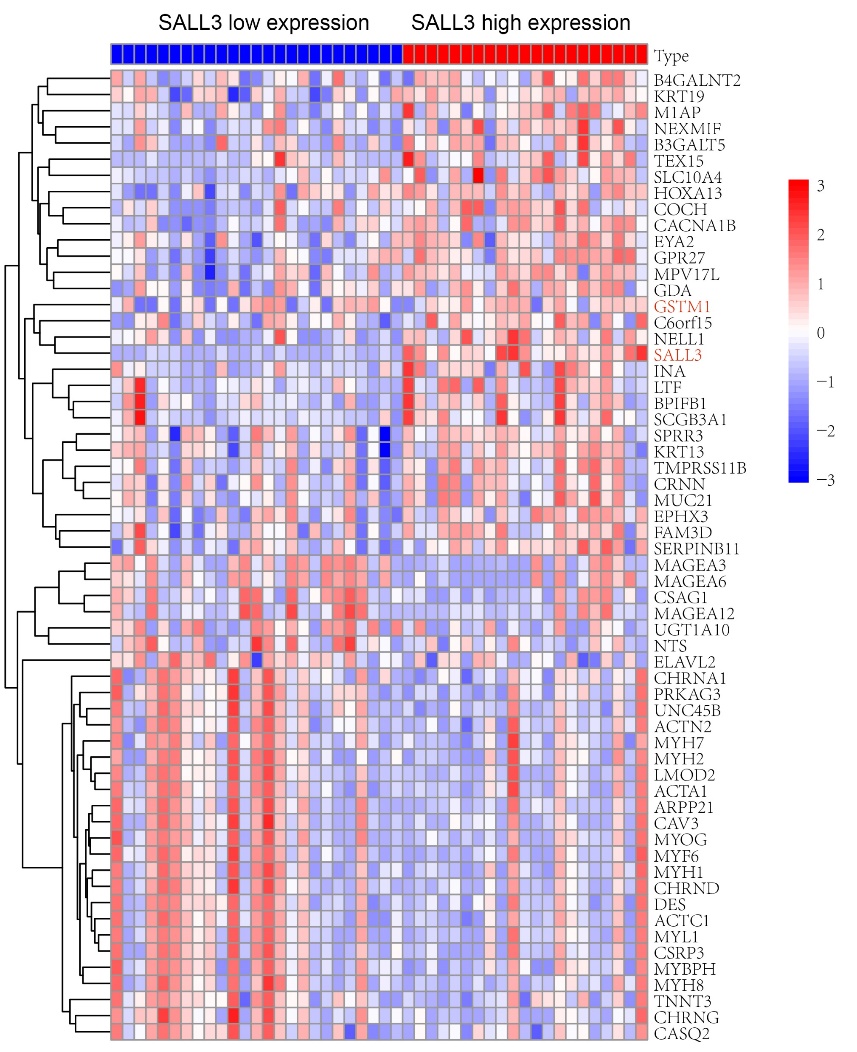


**Figure S4. Bioinformatics analysis for SALL3 gene.** (A) Pathway enrichment analysis of SALL3 in BAA HNSCC. (B) Correlation analysis of gene expression between SALL3 and 29 other top genes with the highest frequencies of CNVs in BAA HNSCC.


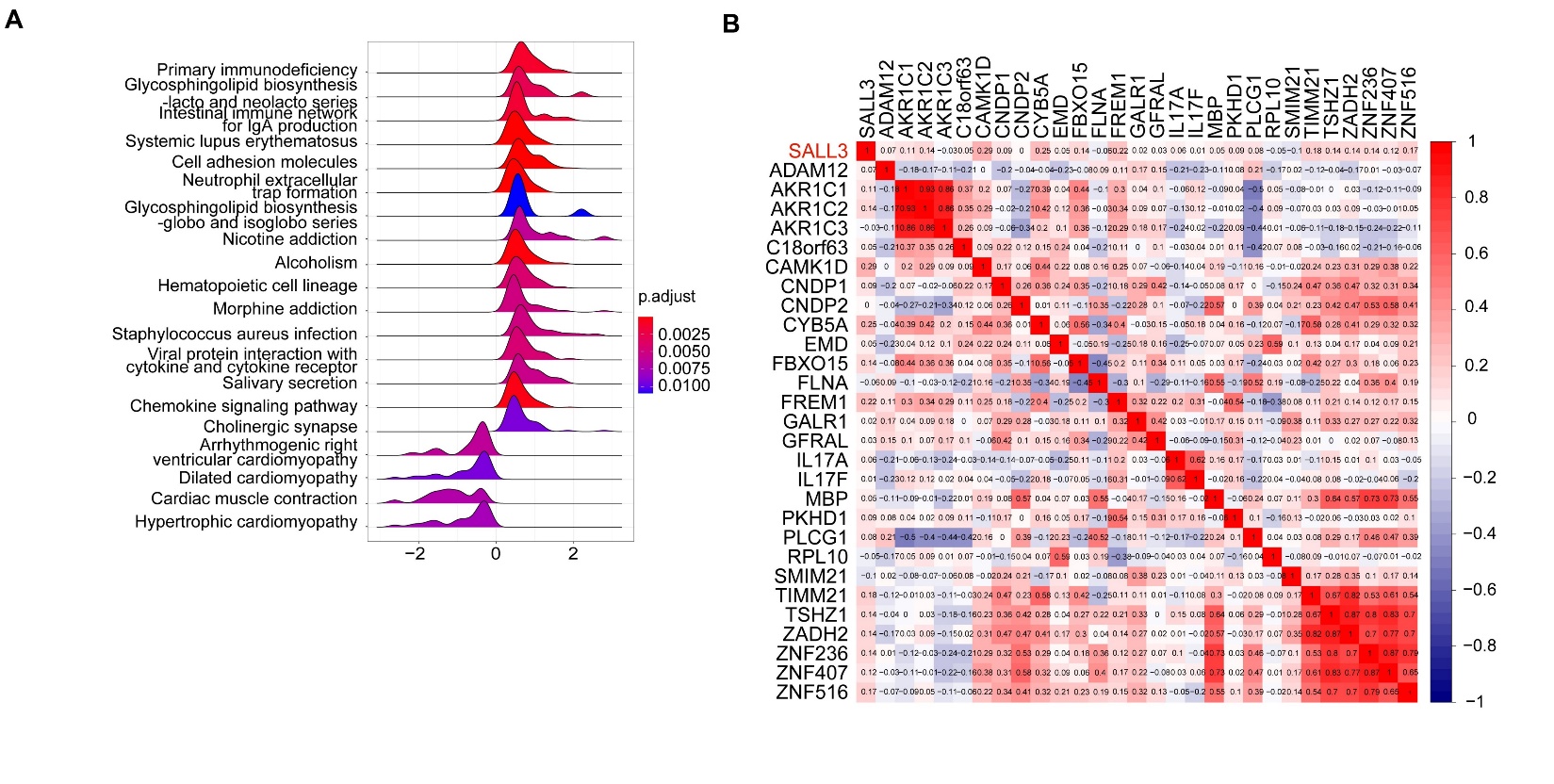


**Figure S5. Correlation analysis of SVIP gene methylation sites and its gene expression in BAA HNSCC.**

**
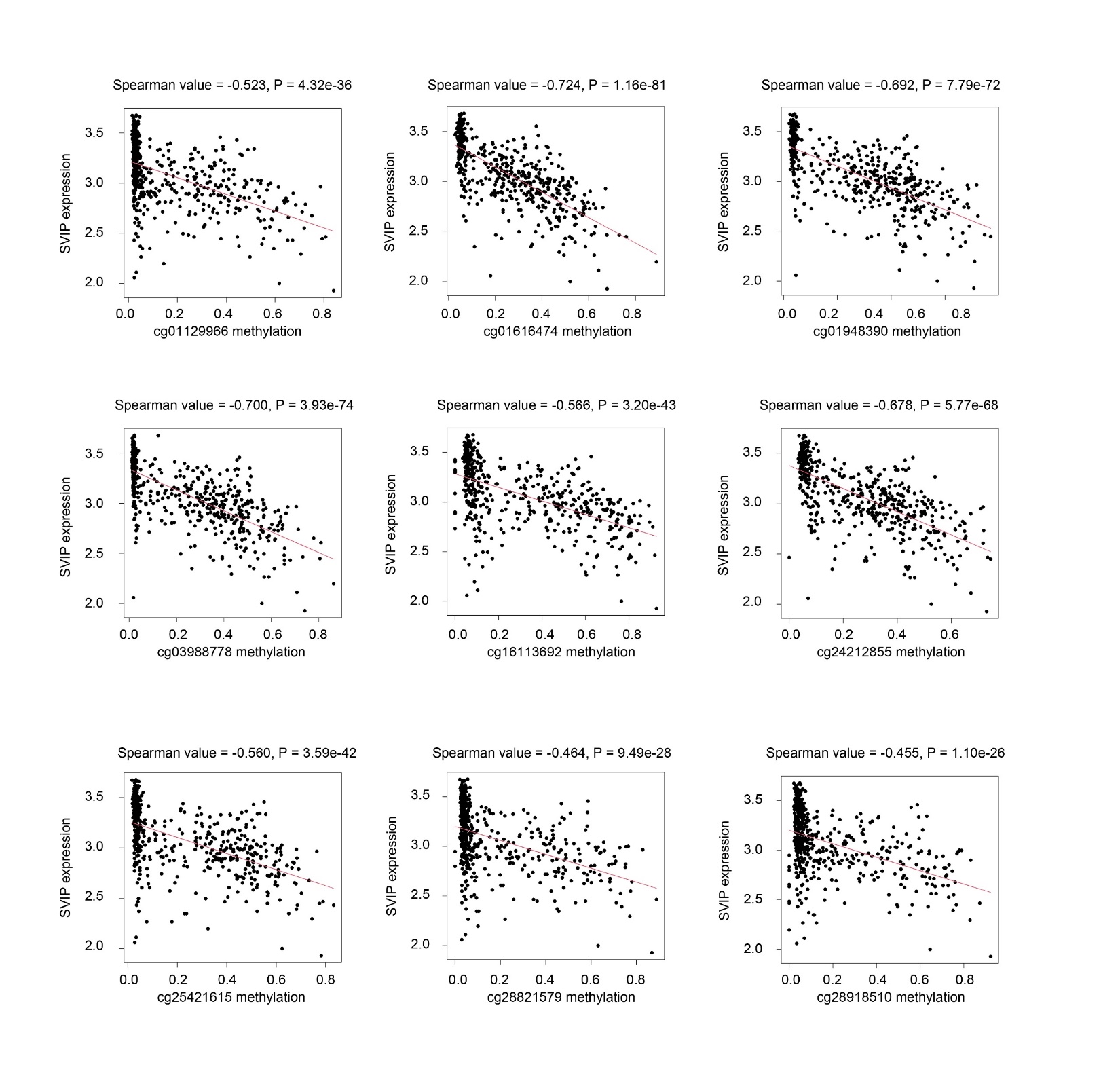
**

**Figure S6.** **Methylation driven genes analysis in BAA HNSCC vs White HNSCC**. (A) Correlation analysis of co-expression between RTP4 and four other methylation driven genes. (B) Correlation between TYMP gene methylation and its expression (left panel), and methylation level analysis of TYMP gene in BAA (green line) and White (orange line) HNSCC (right panel). (C) Correlation between IER5 gene methylation and its expression (left panel), and methylation level analysis of IER5 gene in BAA (green line) and White (orange line) HNSCC (right panel). (D) Correlation between POU3F1 gene methylation and its expression (left panel), and methylation level analysis of POU3F1 gene in BAA (green line) and White (orange line) HNSCC (right panel). (E) Correlation between SIT5 gene methylation and its expression (left panel), and methylation level analysis of SIT5 gene in BAA (green line) and White (orange line) HNSCC (right panel).


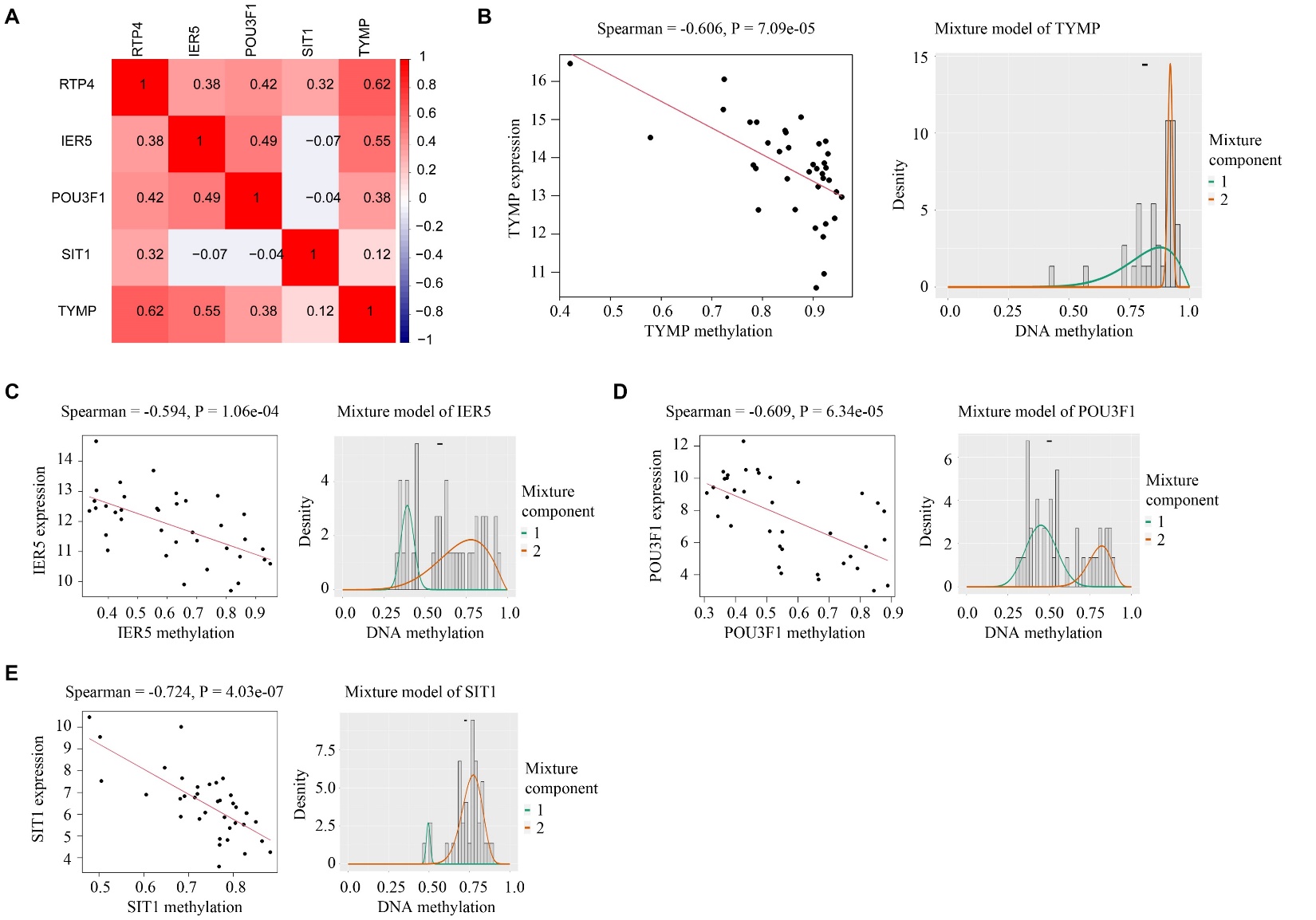


**Figure S7.** **Overall survival analysis for the top 20 upregulated genes in BAAs vs Whites with HNSCC.** ns, not significant, *P<0.05 and **P<0.01.


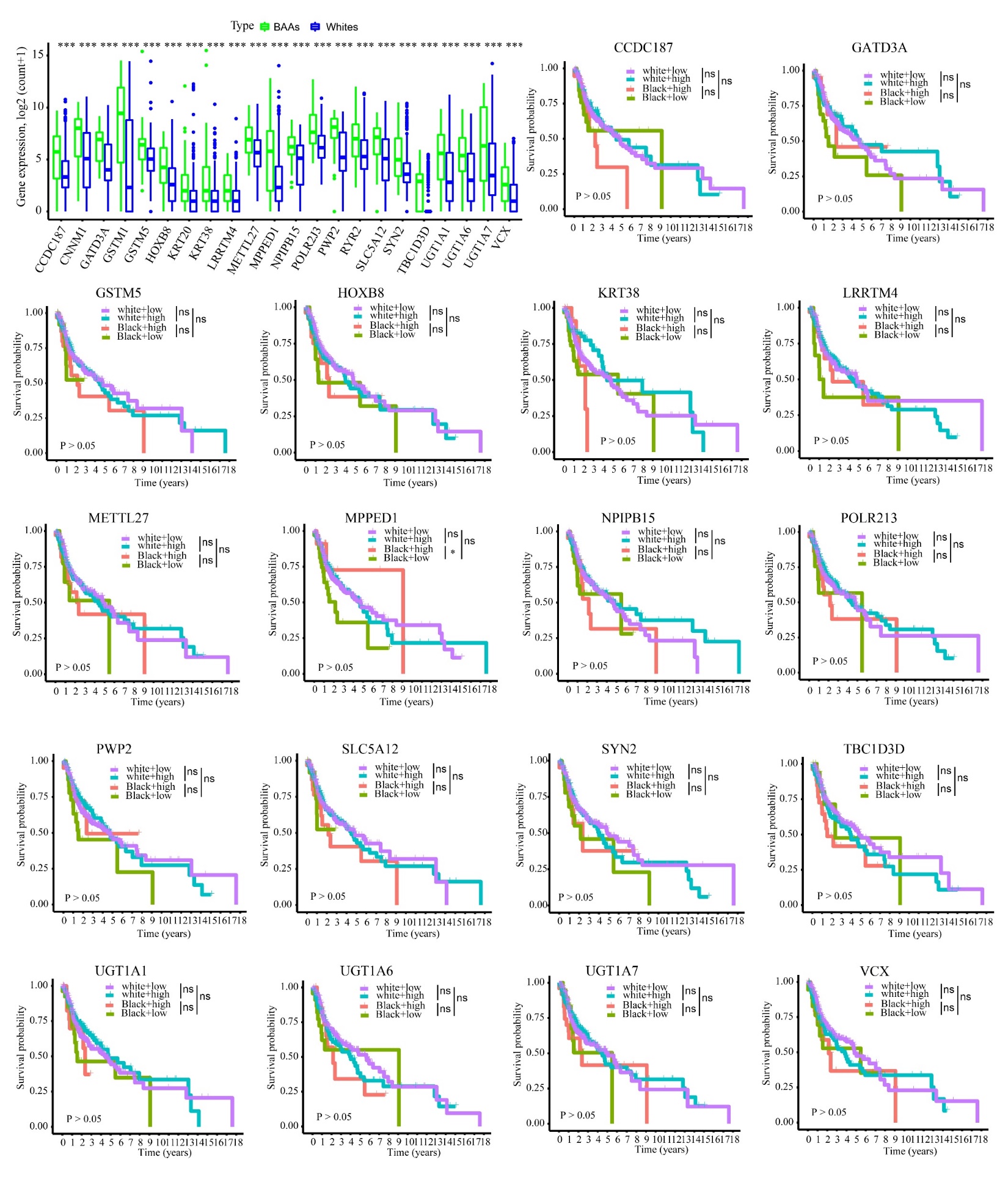


**Figure S8.** **Overall survival analysis for the top 20 downregulated genes in BAAs vs Whites with HNSCC.** ns, not significant, *P<0.05 and **P<0.01.


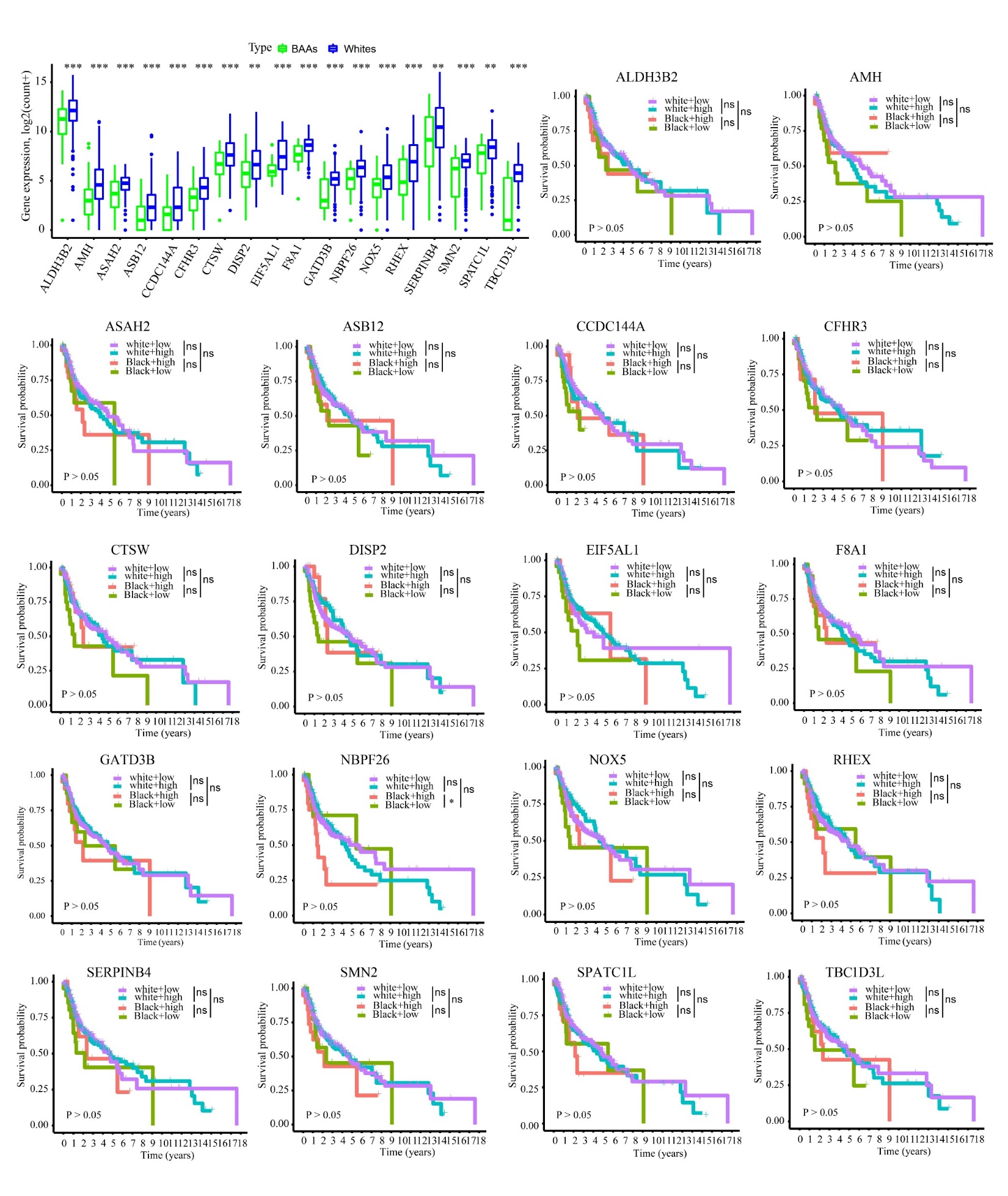


**Figure S9.** **Simultaneous evaluation of the association of GSTM1 expression with the five major clinical characteristics in TCGA HNSCC cohort.** (A) Correlation between GSTM1 expression and the five major clinical characteristics (race, sex, HPV infection status, age and tumor stage) in patients with HNSCC. (B) Comparison of GSTM1 expression in BAA and White patients with HNSCC. (C) Comparison of GSTM1 expression in male and female patients with HNSCC. (D) Comparison of GSTM1 expression in patients with HPV+ HNSCC and HPV- HNSCC. (E) Comparison of GSTM1 expression in HNSCC patients with different ages. (F) Comparison of GSTM1 expression in HNSCC patients with different tumor stages. ns, not significant; ****p*<0.001.


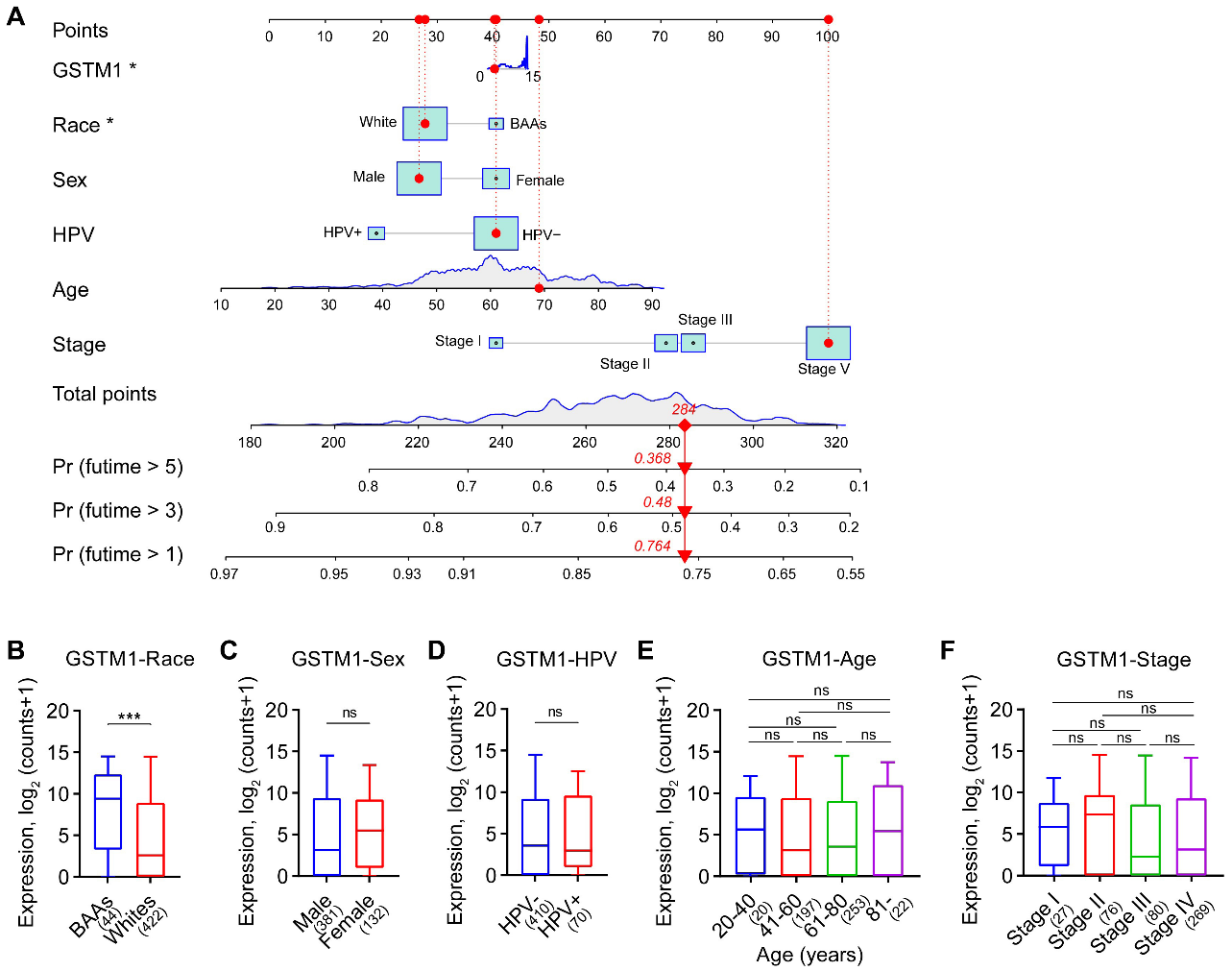


**Figure S10.** **Heatmap showing significantly differentially expressed genes in BAA vs White HNSCC via GSEA.**


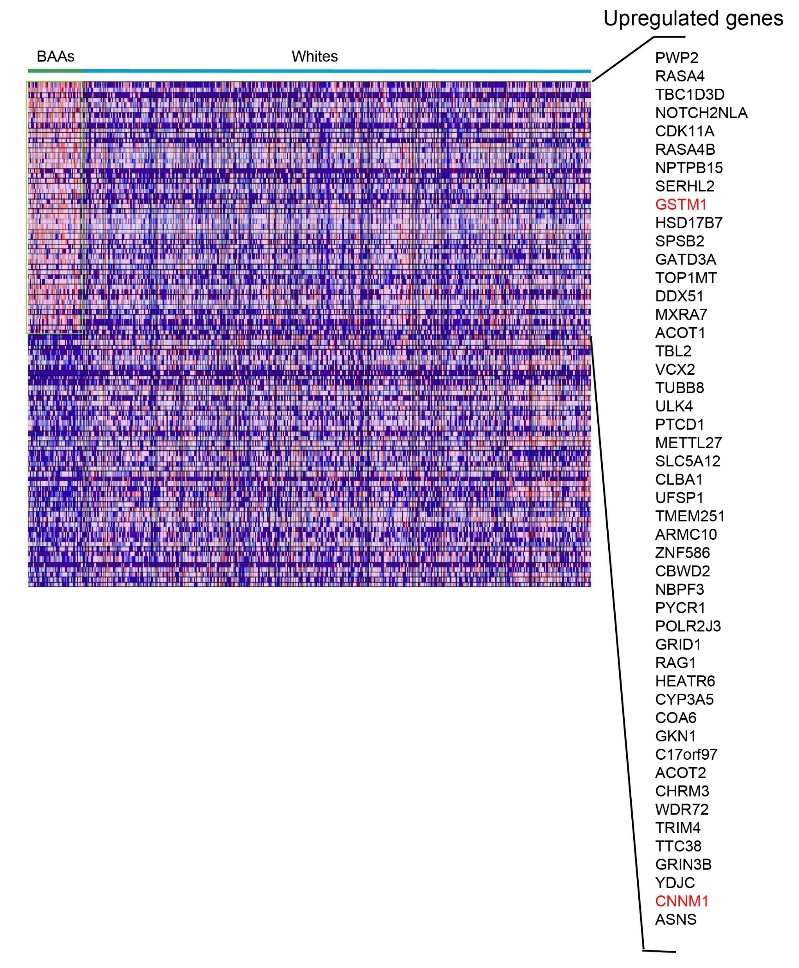


**Table S1. Top 20 mutated genes in BAA HNSCC vs White HNSCC**

| BAAs and Whites | | | | | Whites | | | | | BAAs | | | | |
| --- | --- | --- | --- | --- | --- | --- | --- | --- | --- | --- | --- | --- | --- | --- |
| Gene | TM^1^ | NM^2^ | PS^3^ | PM^4^ | Gene | TM^1^ | NM^2^ | PS^3^ | PM^4^ | Gene | TM^1^ | NM^2^ | PS^3^ | PM^4^ |
| TP53 | 476 | 381 | 598 | 0.637124 | **TP53** | 428 | 341 | 544 | 0.626838 | **TP53** | 48 | 40 | 54 | 0.740741 |
| TTN | 365 | 202 | 598 | 0.337793 | **TTN** | 339 | 186 | 544 | 0.341912 | **TTN** | 26 | 16 | 54 | 0.296296 |
| FAT1 | 161 | 121 | 598 | 0.202341 | **FAT1** | 151 | 113 | 544 | 0.207721 | **MUC16** | 15 | 13 | 54 | 0.240741 |
| CDKN2A | 127 | 119 | 598 | 0.198997 | **CDKN2A** | 117 | 110 | 544 | 0.202206 | **KMT2D** | 16 | 13 | 54 | 0.240741 |
| NOTCH1 | 137 | 113 | 598 | 0.188963 | **NOTCH1** | 126 | 103 | 544 | 0.189338 | **CSMD3** | 17 | 11 | 54 | 0.203704 |
| PIK3CA | 109 | 103 | 598 | 0.172241 | **PIK3CA** | 103 | 99 | 544 | 0.181985 | **LRP1B** | 12 | 11 | 54 | 0.203704 |
| MUC16 | 131 | 95 | 598 | 0.158863 | **CSMD3** | 110 | 82 | 544 | 0.150735 | **NOTCH1** | 11 | 10 | 54 | 0.185185 |
| KMT2D | 119 | 94 | 598 | 0.157191 | **MUC16** | 116 | 82 | 544 | 0.150735 | **CDKN2A** | 10 | 9 | 54 | 0.166667 |
| CSMD3 | 127 | 93 | 598 | 0.155518 | **KMT2D** | 103 | 81 | 544 | 0.148897 | **DNAH5** | 10 | 8 | 54 | 0.148148 |
| SYNE1 | 107 | 82 | 598 | 0.137124 | **SYNE1** | 100 | 75 | 544 | 0.137868 | **UNC13C** | 8 | 8 | 54 | 0.148148 |
| LRP1B | 108 | 82 | 598 | 0.137124 | **LRP1B** | 96 | 71 | 544 | 0.130515 | **FAT1** | 10 | 8 | 54 | 0.148148 |
| PCLO | 88 | 75 | 598 | 0.125418 | **PCLO** | 83 | 70 | 544 | 0.128676 | **NSD1** | 8 | 8 | 54 | 0.148148 |
| DNAH5 | 81 | 66 | 598 | 0.110368 | **NSD1** | 74 | 58 | 544 | 0.106618 | **SYNE1** | 7 | 7 | 54 | 0.12963 |
| NSD1 | 82 | 66 | 598 | 0.110368 | **DNAH5** | 71 | 58 | 544 | 0.106618 | **PKHD1L1** | 7 | 7 | 54 | 0.12963 |
| FLG | 78 | 61 | 598 | 0.102007 | **FLG** | 71 | 57 | 544 | 0.104779 | **FAM135B** | 9 | 7 | 54 | 0.12963 |
| USH2A | 75 | 59 | 598 | 0.098662 | **USH2A** | 70 | 55 | 544 | 0.101103 | **COL11A1** | 8 | 7 | 54 | 0.12963 |
| RYR2 | 73 | 57 | 598 | 0.095318 | **CASP8** | 62 | 54 | 544 | 0.099265 | **RYR2** | 7 | 7 | 54 | 0.12963 |
| CASP8 | 65 | 56 | 598 | 0.093645 | **RYR2** | 66 | 50 | 544 | 0.091912 | **RELN** | 8 | 6 | 54 | 0.111111 |
| DMD | 58 | 51 | 598 | 0.085284 | **XIRP2** | 50 | 47 | 544 | 0.086397 | **LRRC4C** | 7 | 6 | 54 | 0.111111 |
| XIRP2 | 52 | 49 | 598 | 0.08194 | **DMD** | 51 | 46 | 544 | 0.084559 | **RUNX1T1** | 6 | 6 | 54 | 0.111111 |

Note: TM, Total number of mutations; NM, Number of samples with one or more mutations; PS, Profiled Samples; PM, Percentage of samples with one or more mutations.
